# Viral expression of NE/PPE enhances anti-colorectal cancer efficacy of oncolytic adenovirus by promoting TAM M1 polarization to reverse insufficient effector memory/effector CD8^+^ T cell infiltration

**DOI:** 10.1186/s13046-025-03358-y

**Published:** 2025-03-14

**Authors:** Shuo Wang, Lingkai Kong, Linpei Wang, Yan Zhuang, Ciliang Guo, Yuxin Zhang, Huawei Cui, Xiaosong Gu, Junhua Wu, Chunping Jiang

**Affiliations:** 1 State Key Laboratory of Pharmaceutical Biotechnology, Division of Hepatobiliary and Transplantation Surgery, Department of General Surgery Nanjing Drum Tower Hospital, The Affiliated Hospital of Medical School, Nanjing University, Nanjing, 210008 China; 2 Jiangsu Key Laboratory of Molecular Medicine, Medical School, National Institute of Healthcare Data Science at Nanjing University, Nanjing University, Nanjing, 210093 China; 3 Jinan Microecological Biomedicine Shandong Laboratory, Jinan, 250021 China; 4 Department of Hepatobiliary and Pancreatic Surgery, The Second Affiliated Hospital of Fujian Medical University, Quanzhou, Fujian 362000 China

**Keywords:** Oncolytic virus, Neutrophil elastase, Tumor-associated macrophages, Effector memory/Effector CD8 + T cells, Colorectal cancer, Pyroptosis, Tumor microenvironment

## Abstract

**Background:**

Oncolytic adenoviruses are among the most widely utilized oncolytic viruses due to their notable anti-tumor and gene expression capabilities, and modification of ADVs to create armed adenoviruses remains a popular research direction. Nonetheless, immune suppression triggered by ADV and targeted enhancements based on this limitation have been relatively unexplored.

**Methods:**

Flow cytometry was employed to assess immune infiltration in the tumor microenvironment following ADV therapy. Targeted novel recombinant oncolytic viruses, ADV^NE^ and ADV^PPE^, were designed, and their antitumor efficacy, safety, and ability to reshape immune infiltration were evaluated in both subcutaneous tumor models in mice and in vitro experiments. Immune cell depletion assays confirmed the critical role of macrophages. The impact of HMGB1 on macrophage polarization was investigated using shRNA, qRT-PCR, ELISA, and flow cytometry. Furthermore, the importance of TLR4 and its downstream pathways was validated through immunoprecipitation, Western blotting, homozygous knockout mice, and TLR4 inhibitors.

**Results:**

We demonstrated that ADV limits the infiltration of effector memory/effector CD8 + T cells (T_EM_/T_E_) within the tumor microenvironment. To address this, we leveraged the strong capacity of NE or PPE to recruit T_EM_/T_E_ by constructing novel recombinant oncolytic adenoviruses, ADV^NE^ or ADV^PPE^, armed with NE or PPE. These recombinant viruses induce pyroptosis in colorectal cancer cells accompanied by the release of HMGB1. HMGB1 binds to TLR4 on the surface of macrophages, activating the MyD88-NFκB-NLRP3 (ASC) pathway and promoting M1 polarization of TAMs, thereby increasing T_EM_/T_E_ cell infiltration and enhancing antitumor efficacy.

**Conclusions:**

In summary, this study presents the development of the novel oncolytic adenoviruses ADV^NE^ and ADV^PPE^ with enhanced anti-tumor efficacy and provides an in-depth exploration of their specific anti-tumor mechanisms. These findings indicate promising clinical therapeutic prospects and offer new insights for advancing oncolytic adenovirus therapies.

**Supplementary Information:**

The online version contains supplementary material available at 10.1186/s13046-025-03358-y.

## Introduction

Cancer is a major life-threatening disease. According to the latest estimates from the International Agency for Research on Cancer (IARC), nearly 20 million new cancer cases and 9.7 million cancer-related deaths were reported worldwide in 2022. Colorectal cancer ranks third in incidence and second in mortality [1], posing a significant health burden and global public health challenge. Clinical treatment for colorectal cancer typically involves a multimodal approach that combines radiotherapy, chemotherapy, molecular targeted drugs, and surgical resection; however, 54% of patients experience recurrence after comprehensive treatment [2, 3]. This highlights the urgent need to develop new therapeutic approaches.

Immunotherapy represents an innovative strategy in cancer treatment, designed to activate and modulate the patient’s immune system to specifically identify and eliminate cancer cells, with the potential to achieve a curative outcome [4–7]. Various strategies for cancer treatment, including immunostimulatory cytokines, checkpoint inhibitors, chimeric antigen receptor (CAR) T cells, cancer vaccines, and oncolytic virus (OV) immunotherapies, have been proposed to activate the immune system [8]. Oncolytic virus (OV) therapy is a novel, multifunctional cancer treatment that selectively kills tumor cells while activating a systemic immune response [9, 10]. Among these, oncolytic adenoviruses (ADVs) have become one of the most widely used OVs due to the growing number of clinical trials worldwide employing ADVs a gene therapy vectors [11–15]. However, several limitations persist in the clinical application of ADVs, primarily due to restricted self-replication, host antiviral immunity, and the negative immune feedback it induces [16–18]. Thus, by addressing and overcoming the limitations of ADV therapy, we can significantly increase its antitumor efficacy, thereby providing a new therapeutic option for the treatment of colorectal cancer.

Owing to the advantages of ADV therapy—such as safety and reliability, high transduction efficiency, broad cell tropism, high levels of gene expression, and mature production technology—modification of ADVs into novel therapeutic adenoviruses has remained a primary focus in ADV research [19, 20]. Previous studies have engineered ADVs to express proteins such as relaxin, T-cell engagers, and costimulatory molecules [21–23]. Thus, gene modification of ADVs to construct novel therapeutic oncolytic ADVs expressing various proteins is a promising and feasible approach for overcoming the limitations of ADV therapy.

Neutrophil elastase (NE), encoded by the ELANE gene, is a serine protease typically expressed in the primary granules of neutrophils and plays an important role in host defense mechanisms and the coordination of innate immune responses [24]. NE also exhibits unique antitumor properties. Studies have shown that the uptake of NE by breast cancer cells enhances the presentation of tumor-associated antigens by increasing the number of human leukocyte antigen (HLA) class I molecules on the cell surface [25]. Additionally, increased uptake of NE by tumor cells increases the expression of the low-molecular-weight forms of cyclin E, increasing the susceptibility of these cells to cytotoxic T lymphocyte-mediated lysis [26]. Cui and colleagues further established that both NE and porcine pancreatic elastase (PPE, encoded by the CELA1 gene and structurally similar to NE) can selectively kill tumor cells without harming healthy cells and are accompanied by a marked increase in effector memory/effector CD8 + T cells (T_EM_/T_E_) infiltration [27]. Therefore, NE and PPE are safe and effective antitumor proteins that can potentially address insufficient T_EM_/T_E_ infiltration in certain immunotherapies.

In this study, we observed that ADV therapy induces negative immune feedback, leading to reduced T_EM_/T_E_ infiltration within the tumor microenvironment. T_EM_/T_E_, known for their potent cytolytic capabilities and high level of IFN-γ secretion, are considered primary effector cells involved in antitumor responses [28–30]. Given the remarkable capacity of NE and PPE to recruit T_EM_/T_E_, we constructed novel oncolytic ADVs expressing NE or PPE, named ADV^NE^ and ADV^PPE^. We anticipate that NE or PPE expression in these oncolytic ADVs will counteract the insufficient T_EM_/T_E_ infiltration induced by standard ADV treatment, providing new insights into tumor immunotherapy with oncolytic ADVs.

## Results

### ADV therapy in subcutaneous colorectal cancer model mice induces negative immune feedback and a significant reduction in T_EM_/T_E_ infiltration

**Fig. 1:**
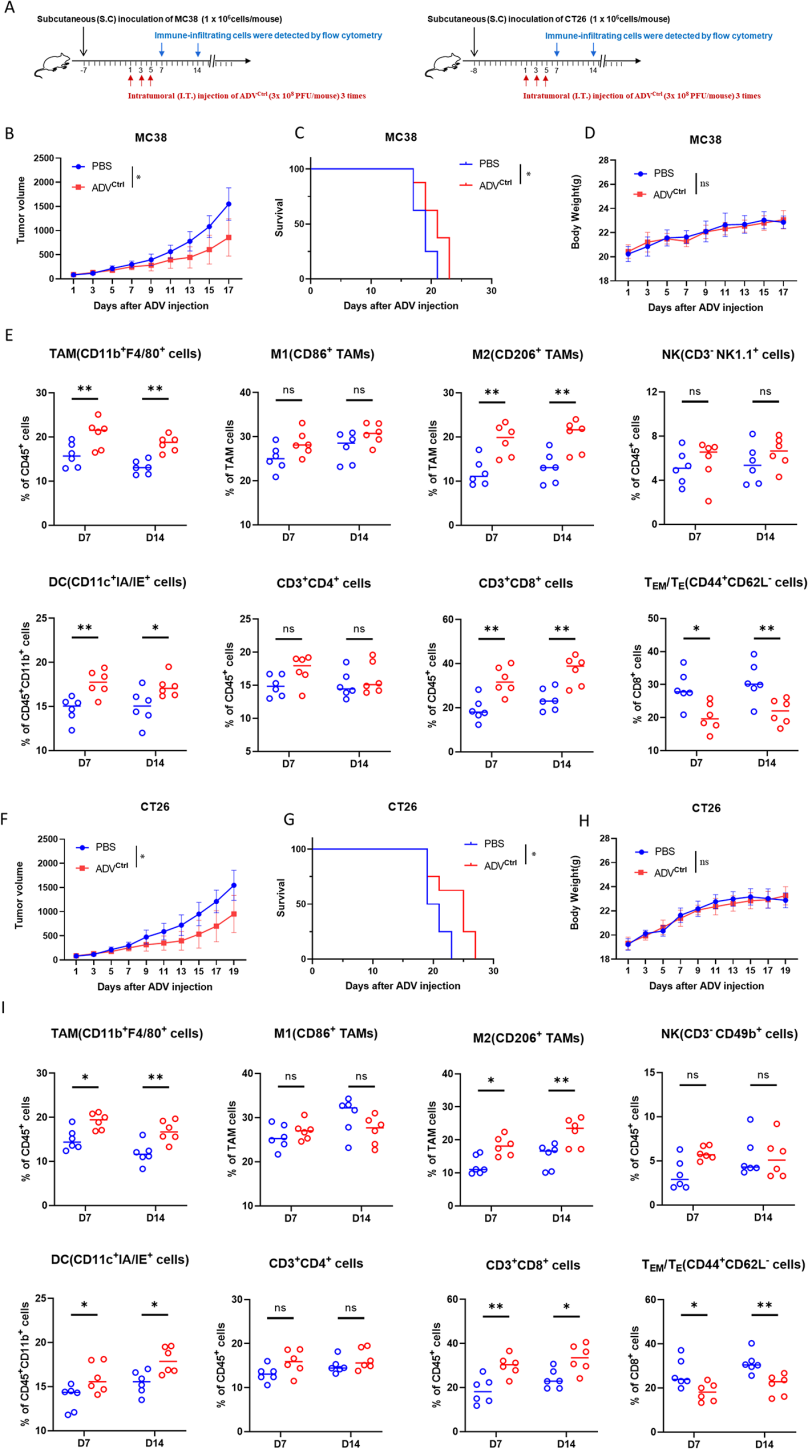
Therapeutic effects of ADV on subcutaneous colorectal cancer model mice and its impact on the tumor microenvironment. (**A**) Schematic of the mouse treatment model. C57BL/6 and BALB/c mice were subcutaneously inoculated with 1 × 10⁶ MC38 or CT26 cells, respectively. When the tumors reached a volume of 50–100 mm³, intratumoral injections of 3 × 10⁸ PFU ADV were administered every other day for a total of three treatments. (**B**) Changes in subcutaneous tumor volume in the MC38 subcutaneous tumor model following treatment with PBS or ADV^Ctrl^ (*n* = 8 mice per group). (**C**) Survival curves of the mice in the different treatment groups (*n* = 8 mice per group). (**D**) Changes in mouse body weight during the treatment period (*n* = 8 mice per group). (**E**) Immune cell infiltration in the tumor microenvironment of MC38 subcutaneous tumors on days 7 and 14 after the first ADV^Ctrl^ treatment (*n* = 6 mice per group). (**F**) Changes in subcutaneous tumor volume in the CT26 subcutaneous tumor model following treatment with PBS or ADV^Ctrl^ (*n* = 8 mice per group). (**G**) Survival curves of the mice in different treatment groups (*n* = 8 mice per group). (**H**) Changes in mouse body weight during the treatment period (*n* = 8 mice per group). (**I**) Immune cell infiltration in the tumor microenvironment of CT26 subcutaneous tumors on days 7 and 14 after the first ADV^Ctrl^ treatment (*n* = 6 mice per group). The data are presented as the means ± standard deviations (SDs). NS, no significant difference; ∗*p* < 0.05, ∗∗*p* < 0.01, ∗∗∗*p* < 0.001, ∗∗∗∗*p* < 0.0001

We utilized MC38 and CT26 murine subcutaneous tumor models to evaluate the therapeutic efficacy of ADV^Ctrl^ (Fig. 1A). The results indicated that ADV^Ctrl^ treatment notably inhibited tumor growth and prolonged survival time (Fig. 1B-C) without significantly affecting body weight (Fig. 1D). To assess changes in the immune microenvironment, we collected mouse tissues on days 7 and 14 following the first ADV^Ctrl^ treatment for flow cytometry analysis. Compared with the PBS-treated group, the ADV^Ctrl^-treated group showed no significant changes in the proportions of intratumoral CD4^+^ T cells, M1 macrophages, or natural killer (NK) cells. Although there was a significant increase in dendritic cell (DC) and CD8^+^ T-cell infiltration, we observed a concurrent significant increase in M2 macrophages and a marked decrease in T_EM_/T_E_ infiltration (Fig. 1E). Similar therapeutic effects (Fig. 1F-H) and immune microenvironment profiles (Fig. 1I) were also observed in the CT26 murine subcutaneous tumor model. These findings suggest that while ADV^Ctrl^ possesses notable antitumor properties, it also induces negative immune feedback characterized by a significant reduction in T_EM_/T_E_ infiltration and an increase in M2 macrophage infiltration.

### The recombinant oncolytic ADVs ADV^NE^ and ADV^PPE^ significantly increase oncolytic activity and induce pyroptosis in colorectal cancer cells, as well as the release of HMGB1

**Fig. 2:**
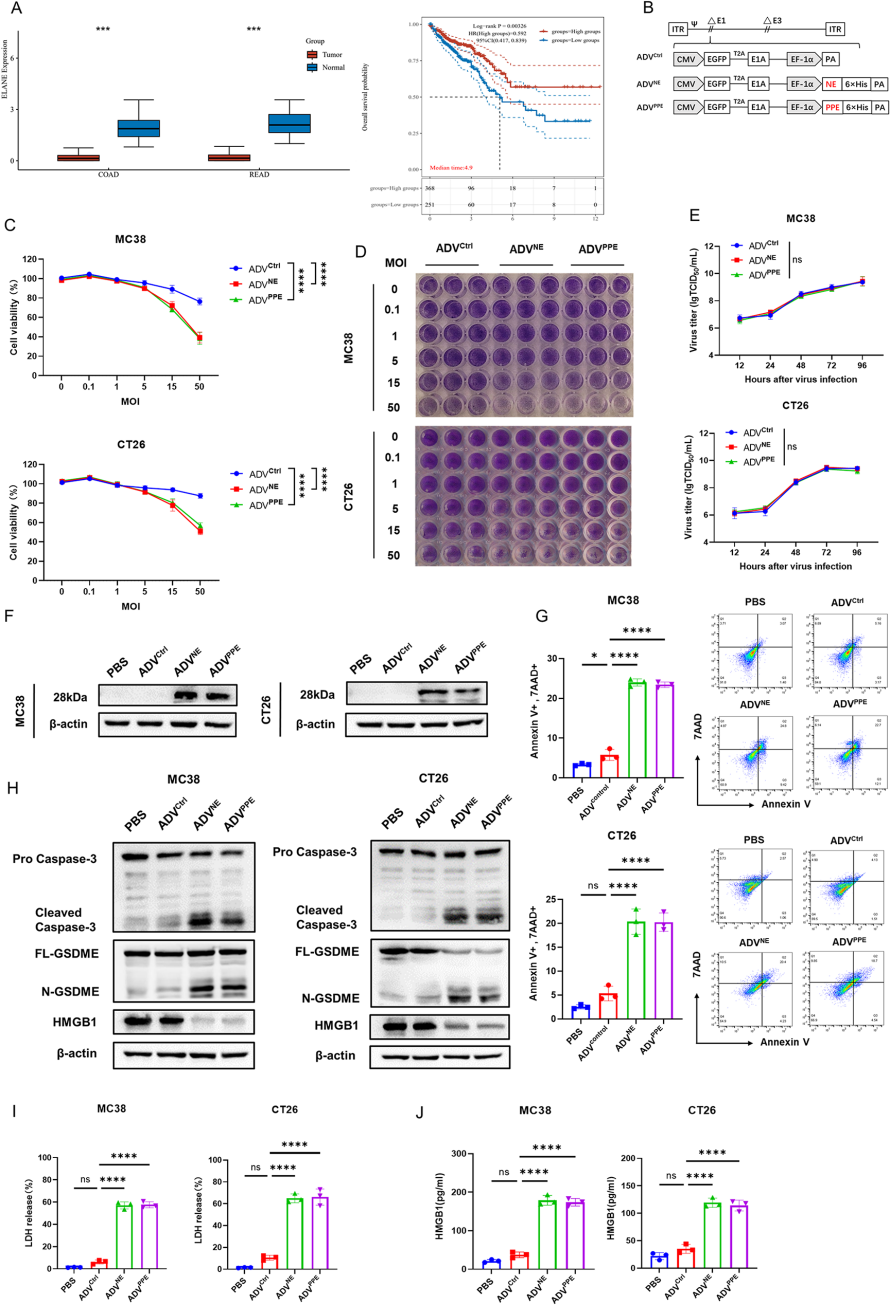
Construction and characterization of ADV^NE^ and ADV^PPE^ and investigation of their in vitro antitumor mechanisms. (**A**) Differences in ELANE gene expression between tumor and adjacent normal tissues and overall survival of colorectal cancer patients with high versus low ELANE expression were analyzed using the TCGA database. (**B**) Schematic structure of the recombinant oncolytic ADVs ADV^NE^ and ADV^PPE^. (**C**-**D**) To evaluate the oncolytic activity of the recombinant ADVs, MC38 and CT26 cells were infected with ADV^Ctrl^, ADV^NE^, or ADV^PPE^ at different multiplicities of infection (MOIs). Cytotoxicity was assessed 48 h post infection via a CCK-8 assay (**C**) and crystal violet staining (**D**) (*n* = 3 biological replicates). (**E**) To assess the replication capacity of oncolytic ADVs, MC38 and CT26 cells were infected at an MOI of 1, and viral titers were measured at 12 h, 24 h, 48 h, 72 h, and 96 h post infection via a TCID_50_ assay (*n* = 3 biological replicates). (**F**) Expression of NE and PPE in MC38 and CT26 cells infected with different ADVs, as detected by Western blotting with an anti-His antibody. (**G**) MC38 and CT26 cells were infected with different viruses, and apoptosis was assessed 48 h later via Annexin-V and 7AAD staining, followed by flow cytometry (*n* = 3 biological replicates). (**H**) Levels of cleaved Caspase-3, N-GSDME, and HMGB1 in MC38 and CT26 cells after infection with different ADVs, as detected by Western blotting. (**I**-**J**) Following infection of MC38 and CT26 cells with different viruses for 48 h, LDH release was determined using an LDH Release Assay Kit (**I**), and HMGB1 levels in the supernatant were measured via ELISA (**J**) (*n* = 3 biological replicates). The data are presented as the means ± SDs. NS, no significant difference; ∗*p* < 0.05, ∗∗*p* < 0.01, ∗∗∗*p* < 0.001, ∗∗∗∗*p* < 0.0001

Reports indicate that NE and PPE exhibit strong selective cytotoxicity against various tumor cell lines and significantly increase T_EM_/T_E_ infiltration within tumor tissues. This property may effectively address the insufficient T_EM_/T_E_ infiltration observed with ADV monotherapy. Additionally, our analysis of data obtained from The Cancer Genome Atlas (TCGA) database revealed that ELANE gene expression is higher in normal tissues than in tumor tissues and that colorectal cancer patients with high ELANE expression have a better prognosis (Fig. 2A). These findings suggest that NE is a promising candidate for generating oncolytic ADVs, which led us to construct NE- and PPE-expressing oncolytic adenoviruses, designated ADV^NE^ and ADV^PPE^ (Fig. 2B). Given the selective cytotoxicity of NE and PPE toward tumor cells, we assessed the oncolytic capacities of these recombinant ADVs in MC38 and CT26 cell lines via CCK-8 assays (Fig. 2C) and crystal violet staining (Fig. 2D). The results demonstrated that ADV^NE^ and ADV^PPE^ exhibited significantly stronger oncolytic effects than did ADV^Ctrl^. Furthermore, TCID_50_ assays revealed that all three viruses had comparable replication capacities (Fig. 2E), and Western blotting confirmed that the recombinant ADVs expressed the NE and PPE proteins within tumor cells (Fig. 2F).

To evaluate the effects of ADV^Ctrl^, ADV^NE^, and ADV^PPE^ on nontumor cells, we examined their effects on HEK-293T cells. The results indicated that the expression of NE or PPE by the recombinant viruses did not enhance the effect of ADV on HEK-293T cell viability (Fig. S1A) or affect ADV replication within HEK-293T cells (Fig. S1B).

According to Cui et al., NE and PPE proteins can increase the levels of cleaved Caspase-3 in tumor cells, thereby inducing apoptosis [27]. We therefore assessed the apoptosis-inducing ability of each virus. Compared with the ADV^Ctrl^-treated group, the ADV^NE−^ and ADV^PPE^-treated groups presented a significant increase in the proportion of Annexin-V and 7AAD double-positive cells (Fig. 2G). Western blot analysis further revealed that the levels of cleaved Caspase-3 and N-GSDME in tumor cells were markedly elevated after infection with ADV^NE^ or ADV^PPE^, whereas the HMGB1 levels decreased (Fig. 2H) and LDH release increased (Fig. 2I), all of which are indicators of pyroptosis [31]. Additionally, the results of Western blot analysis excluded the possibility of necroptosis (Fig. S2). Thus, we propose that NE expressed by ADV^NE^ and PPE expressed by ADV^PPE^ activate Caspase-3 in colorectal cancer cells, which in turn cleaves GSDME to release N-GSDME, thereby inducing pyroptosis and increasing HMGB1 release (Fig. 2J). These findings demonstrate that we successfully constructed novel oncolytic ADVs, ADV^NE^ and ADV^PPE^, which are capable of expressing NE or PPE. The NE- and PPE-expressing oncolytic ADVs significantly induced pyroptosis and had enhanced oncolytic activity in colorectal cancer cells without affecting ADV replication in tumor cells. Additionally, the expression of NE or PPE by these novel oncolytic ADVs did not affect nontumor cells, such as HEK-293T cells.

### The novel therapuetic oncolytic adenoviruses ADV^NE^ and ADV^PPE^ exhibited significantly increased antitumor efficacy in a mouse colorectal cancer model

**Fig. 3:**
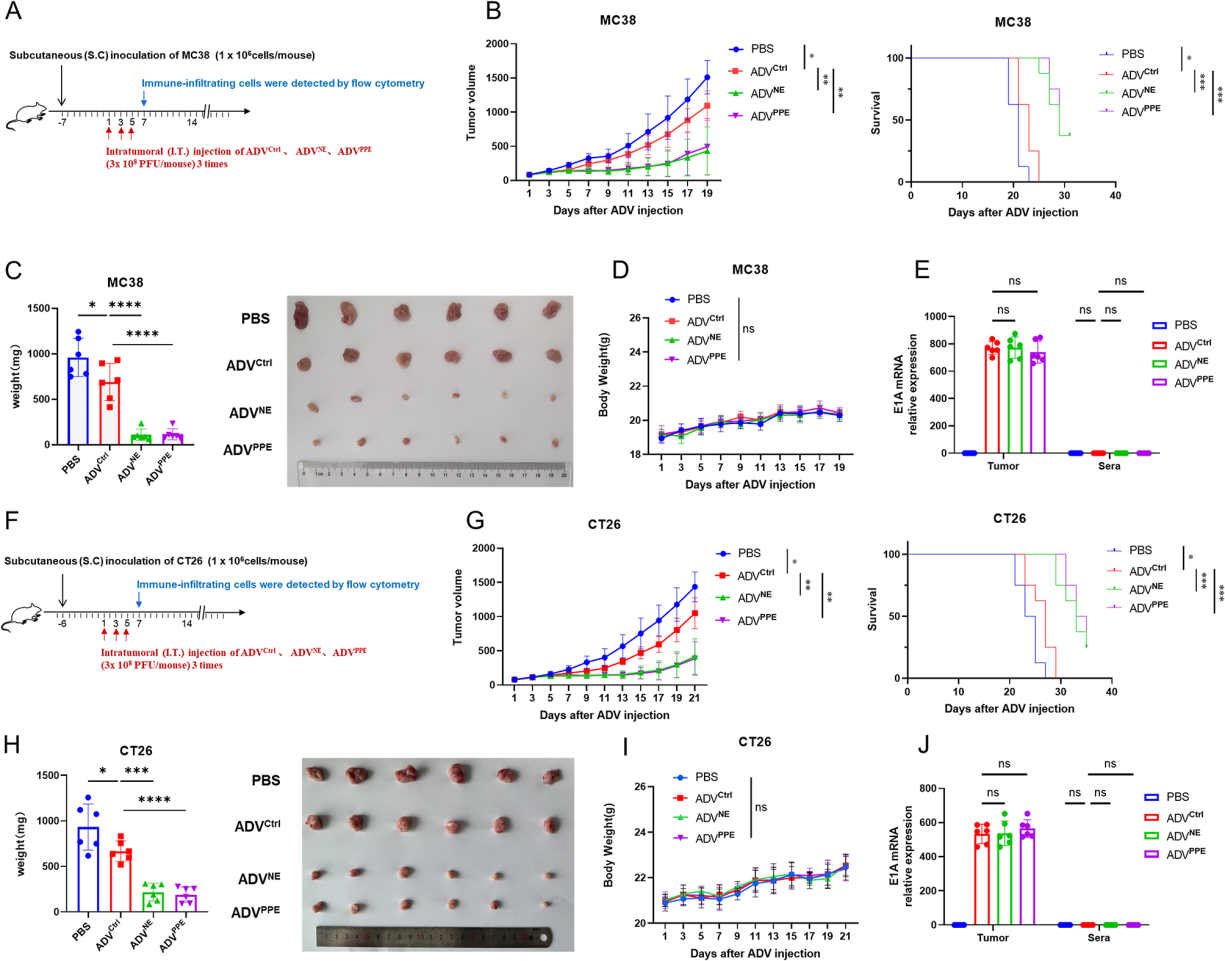
Antitumor effects of the recombinant oncolytic adenoviruses ADV^NE^ and ADV^PPE^ in a colorectal cancer murine model. (**A**) Schematic of the mouse model. C57BL/6 mice were subcutaneously inoculated with 1 × 10⁶ MC38 cells. When the tumors reached a volume of 50–100 mm³, intratumoral injections of 3 × 10⁸ PFU of adenovirus were administered every other day for a total of three treatments. (**B**) Changes in subcutaneous tumor volume and survival in the MC38 subcutaneous tumor model mice following treatment with PBS or ADV^Ctrl^ were recorded every two days (*n* = 8 mice per group). (**C**) Tumor weights were measured prior to harvest and analysis via flow cytometry (*n* = 6 mice per group). (**D**) Mouse body weights were measured every two days during the survival observation period (*n* = 8 mice per group). (**E**) Blood and tumor tissues were collected on day 7 after the first virus treatment. Tumor samples (100 mg) were homogenized in 200 µl of PBS. The supernatant and serum were separated via centrifugation. qPCR was used to measure the expression of the ADV-specific gene E1A, indicating viral distribution (*n* = 3 biological replicates). (**F**-**J**) BALB/c mice were subcutaneously inoculated with 1 × 10⁶ CT26 cells, and the experiments described in **A**-**E** were repeated. The data are presented as the means ± SDs. NS, no significant difference; ∗*p* < 0.05, ∗∗*p* < 0.01, ∗∗∗*p* < 0.001, ∗∗∗∗*p* < 0.0001

To evaluate the antitumor effects of the recombinant ADVs, we established an MC38 subcutaneous tumor mouse model and treated the mice with different ADVs (Fig. 3A). The results revealed that the tumor diameters in the ADV^NE^ and ADV^PPE^ treatment groups were significantly smaller than those in the ADV^Ctrl^ group (*p* < 0.001), and the survival times were notably longer (*p* < 0.05) (Fig. 3B). In each of the ADV^NE^ and ADV^PPE^ treatment groups, three mice achieved complete tumor remission. Upon reinoculation of MC38 cells into these cured mice, no new solid tumors formed, indicating lasting immunity. Tumor weights, which were measured before flow cytometry analysis, further confirmed the reduced tumor burden in the ADV^NE^ and ADV^PPE^ groups (Fig. 3C). Observations of body weight changes revealed no significant impact on mouse weight across the different virus treatments (Fig. 3D). To determine viral distribution, qPCR was performed to measure the expression of the ADV-specific gene E1A. The results revealed no significant differences in the viral content within the tumor homogenates among the ADV^Ctrl^, ADV^NE^, and ADV^PPE^ groups, and no corresponding ADVs were detected in the mouse serum (Fig. 3E). Similar results were obtained in the CT26 subcutaneous tumor model (Fig. 3F-J), confirming that the recombinant viruses ADV^NE^ and ADV^PPE^ demonstrate not only greater antitumor efficacy than ADV^Ctrl^ but also the same safety profile as ADV^Ctrl^.

### ADV^NE^ and ADV^PPE^ increase the antitumor immunotherapy of advs by promoting TAM M1 polarization and increasing T_EM_/T_E_ infiltration

**Fig. 4:**
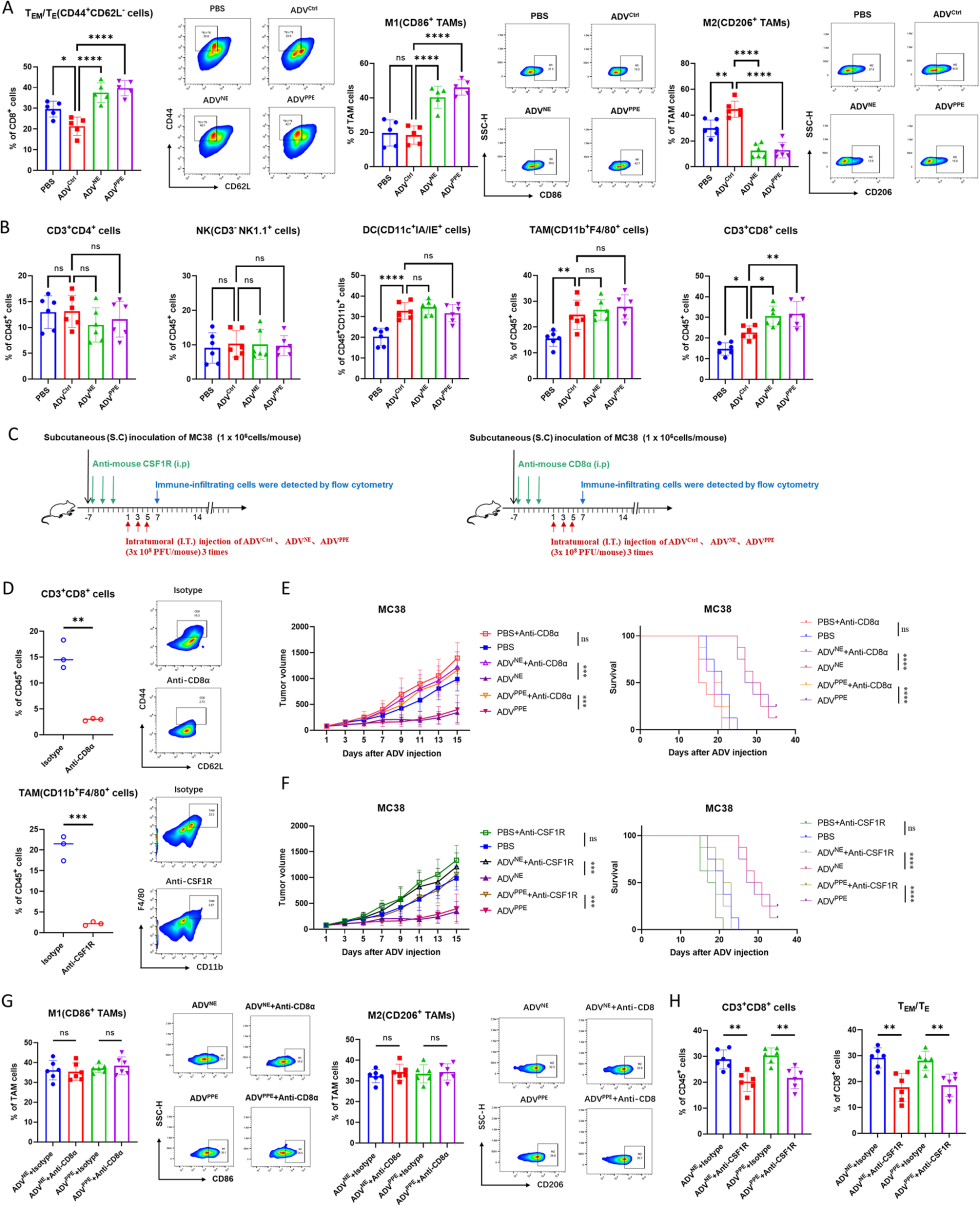
Effects of ADV^NE^ and ADV^PPE^ on Macrophage M1 Polarization and T_EM_/T_E_ Infiltration. (**A**) C57BL/6 mice were subcutaneously inoculated with 1 × 10⁶ MC38 cells. When the tumors reached a volume of 50–100 mm³, intratumoral injections of 3 × 10⁸ PFU of ADV were administered every other day for a total of three treatments. On day 7 after the first virus treatment, flow cytometry was used to assess the proportions of M1 and M2 macrophages and the infiltration of T_EM_/T_E_ in the tumor microenvironment (*n* = 6 mice per group). (**B**) Infiltration of TAMs, NK cells, DCs, CD4^+^ T cells, and CD8^+^ T cells in the tumor microenvironment of mice following different virus treatments (*n* = 6 mice per group). (**C**) Schematic of the immune cell depletion experiment. C57BL/6 mice were subcutaneously inoculated with 1 × 10⁶ MC38 cells. Starting the next day, the mice received intraperitoneal injections of 500 µg of anti-CD8α or anti-CSF1R every other day to deplete CD8^+^ T cells or macrophages, respectively. When the tumors reached 50–100 mm³, intratumoral injections of 3 × 10⁸ PFU of adenovirus were administered every other day for a total of three treatments. On day 7 after the first virus treatment, flow cytometry was used to evaluate immune cell infiltration into the tumor microenvironment. (**D**) Efficiency of macrophage and CD8^+^ T-cell depletion in the tumor tissues of MC38 tumor-bearing mice one week after treatment with anti-CD8α and anti-CSF1R antibodies, as assessed by flow cytometry. (**E**) Subcutaneous tumor volume and survival of mice in different treatment groups after CD8^+^ T-cell depletion (*n* = 8 mice per group). (**F**) Subcutaneous tumor volume and survival of mice in different treatment groups after macrophage depletion (*n* = 8 mice per group). (**G**) Infiltration of M1-like and M2-like macrophages in the tumor microenvironment after CD8^+^ T-cell depletion (*n* = 6 mice per group). (**H**) Infiltration of CD8^+^ cells and T_EM_/T_E_ cells in the tumor microenvironment after macrophage depletion (*n* = 6 mice per group). The data are presented as the means ± SDs. NS, no significant difference; ∗*p* < 0.05, ∗∗*p* < 0.01, ∗∗∗*p* < 0.001, ∗∗∗∗*p* < 0.0001

To investigate the mechanisms underlying the antitumor effects of the recombinant oncolytic ADVs, we conducted flow cytometry to examine the immune microenvironment in the MC38 subcutaneous tumors. The results showed that ADV^NE^ and ADV^PPE^ treatments effectively abrogated the insufficient T_EM_/T_E_ infiltration observed in the ADV^Ctrl^ treatment group. Additionally, compared with the ADV^Ctrl^ treatment, treatment with ADV^NE^ or ADV^PPE^ increased the infiltration of M1 macrophages and CD8^+^ T cells within the tumor microenvironment while reducing the proportion of M2 macrophages. (Fig. 4A -B). However, there were no significant differences among the three virus treatments in the proportions of TAMs, NK cells, DCs, or CD4^+^ T cells within the tumor microenvironment (Fig. 4B). Furthermore, similar observations were made in the CT26 subcutaneous tumor model (Fig. S3).

These results suggest that the recombinant viruses primarily affect macrophages and CD8^+^ T cells in the tumor microenvironment. To clarify their roles, we performed depletion experiments on macrophages and CD8^+^ T cells in the MC38 subcutaneous tumor model (Fig. 4C-D). Observations of tumor volume and mouse survival time revealed that the depletion of either macrophages or CD8^+^ T cells abrogated the enhanced therapeutic efficacy of ADV^NE^ and ADV^PPE^. (Fig. 4E-F).

CD8^+^ T cells can influence macrophage polarization through cytokine secretion, while macrophages can affect the antitumor efficacy of CD8^+^ T cells via chemokine release [32–34]. To elucidate the specific interactions between macrophages and CD8^+^ T cells during recombinant virus treatment, we established MC38 subcutaneous tumors, grouped the mice according to the depletion of macrophages or CD8^+^ T cells, and treated them with ADV^NE^ or ADV^PPE^. Flow cytometry analysis of immune infiltration in tumor tissues revealed that depletion of CD8^+^ T cells did not affect the polarization of macrophages in the ADV^NE^ and ADV^PPE^ treatment groups (Fig. 4G). However, the depletion of macrophages abrogates the increases in CD8^+^ T cells and T_EM_/T_E_ cells induced by the recombinant viruses (Fig. 4H).

These findings suggest that the enhanced antitumor effects of the novel recombinant viruses ADV^NE^ and ADV^PPE^ depend on the presence of both macrophages and CD8^+^ T cells. Furthermore, macrophages in the tumor microenvironment exert a greater influence on CD8^+^ T-cell and T_EM_/T_E_ infiltration than do CD8^+^ T cells on macrophage polarization. Therefore, we conclude that NE and PPE expressed by recombinant ADVs promote TAM polarization toward the M1 phenotype, thereby increasing CD8^+^ T-cell and T_EM_/T_E_ infiltration in the TME and increasing the antitumor efficacy of ADV^Ctrl^.

### HMGB1 released by tumor cells treated with ADV^NE^ or ADV^PPE^ induces macrophage M1 polarization and increases T_EM_/T_E_ infiltration

**Fig. 5:**
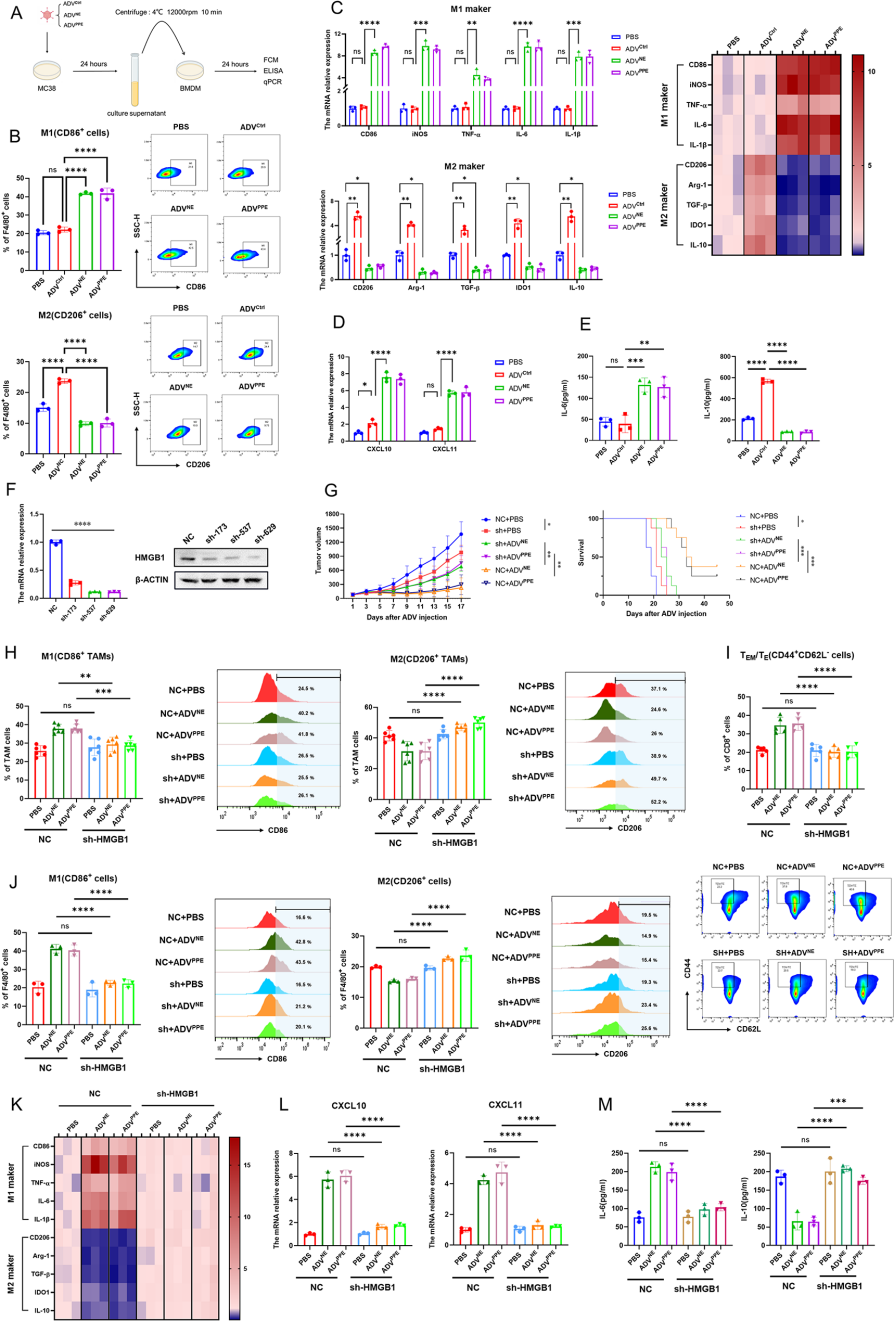
Effects of HMGB1 Release by Tumor Cells Treated with ADV^NE^ or ADV^PPE^ on Macrophage Polarization and T_EM_/T_E_ Infiltration. (**A**) Schematic of the in vitro experimental model shown in 5B-5E: MC38 cells were treated with PBS, ADV^Ctrl^, ADV^NE^, or ADV^PPE^, and after 24 h, the tumor cell supernatant was collected to stimulate BMDMs. Flow cytometry, qPCR, and ELISA were performed 24 h after stimulation. (**B**) Polarization state of BMDMs after 24 h of stimulation with supernatants from different treatment groups, as assessed by flow cytometry (*n* = 3 biological replicates). (**C**) qPCR analysis of gene expression in BMDMs for CD86, iNOS, TNF-α, IL-6, IL-1β, Arg-1, CD206, TGF-β, IDO1, and IL-10, with expression levels presented as a heatmap (*n* = 3 biological replicates). (**D**) qPCR analysis of M1 macrophage-related chemokines CXCL10 and CXCL11 in BMDMs (*n* = 3 biological replicates). (**E**) ELISA of IL-6 and IL-10 levels in the supernatants of BMDMs after 24 h of stimulation with different treatments (*n* = 3 biological replicates). (**F**) Knockdown of HMGB1 in MC38 cells via lentivirus carrying knockout constructs; qPCR and Western blotting were used to assess the knockdown efficiency. The shRNA construct with the highest knockdown efficiency, sh-629, was used to establish the shHMGB1-MC38 cell line. (**G**) Subcutaneous tumor models were established in mice via the use of shHMGB1-MC38 and NC-MC38 cells, followed by treatment with PBS, ADV^NE^, or ADV^PPE^. Subcutaneous tumor volume and survival were monitored every two days (*n* = 8 mice per group). (**H**-**I**) Subcutaneous tumor models were reestablished using shHMGB1-MC38 and NC-MC38 cells, followed by various treatments. On day 7 after the first virus treatment, flow cytometry was used to assess the infiltration of M1 macrophages, M2 macrophages (**H**), and T_EM_/T_E_ cells (**I**) in tumor tissues (*n* = 6 mice per group). (**J**) MC38 cells (shHMGB1-MC38 and NC-MC38) were treated with PBS, ADV^NE^, or ADV^PPE^, and after 24 h, the tumor cell supernatant was collected to stimulate BMDMs. After an additional 24 h, the polarization of the BMDMs was assessed via flow cytometry (*n* = 3 biological replicates). (**K**-**M**) BMDMs were stimulated with supernatants from the various treatment groups for 24 h, followed by experiments as described in **C**-**E** (*n* = 3 biological replicates). The data are presented as the means ± SDs. NS, no significant difference; ∗*p* < 0.05, ∗∗*p* < 0.01, ∗∗∗*p* < 0.001, ∗∗∗∗*p* < 0.0001

Since the increased M1 polarization of macrophages in the tumor microenvironment can promote T_EM_/T_E_ infiltration, we further investigated the specific mechanisms through which the recombinant viruses ADV^NE^ and ADV^PPE^ induce M1 macrophage polarization. Growing evidence suggests that secreted proteins play a key role in regulating macrophage polarization [35–38]. To determine whether the M1 polarization observed in this study was induced by factors released by tumor cells into the culture medium, we treated bone marrow-derived macrophages (BMDMs) with different culture media (Fig. 5A). Flow cytometry revealed that culture supernatant from recombinant virus-treated tumor cells significantly promoted M1 polarization but inhibited M2 polarization in macrophages (Fig. 5B). Correspondingly, the qPCR results revealed a significant increase in the mRNA expression levels of the M1-related genes CD86 and iNOS in BMDMs, along with a marked decrease in the mRNA expression of the M2-related genes Arg-1 and CD206. Moreover, the levels of proinflammatory cytokines (TNF-α, IL-6, and IL-1β) were significantly increased, whereas those of immunosuppressive factors (such as TGF-β, IDO1, and IL-10) were markedly decreased (Fig. 5C). Additionally, the mRNA expression levels of M1-related chemokines (CXCL10 and CXCL11) were significantly increased (Fig. 5D). The ELISA results revealed that the culture supernatant from recombinant virus-treated tumor cells significantly increased IL-6 secretion and decreased IL-10 secretion in BMDMs (Fig. 5E). Similarly, when RAW264.7 cells were stimulated with different culture supernatants, consistent results were observed(Fig. S4A-E).

Reports suggest that HMGB1 can induce M1 macrophage polarization [39–41]. Furthermore, we observed a significant increase in HMGB1 released into the culture supernatant by colorectal cancer cells treated with recombinant adenoviruses compared to the ADV^Ctrl^ group (Fig. 2J). To confirm the role of HMGB1 in promoting M1 macrophage polarization, we transfected MC38 with lentivirus containing HMGB1 knockout constructs to knock down HMGB1 in MC38 (Fig. 5F) and established corresponding subcutaneous tumor models in mice. We found that while HMGB1 knockdown slightly slowed tumor growth, it also abrogated the therapeutic efficacy of the recombinant viruses (Fig. 5G). Flow cytometry analysis of immune infiltration in tumor tissues revealed that HMGB1 knockdown abrogated the increase in the proportion of M1 macrophages and decrease in the proportion of M2 macrophages induced by ADV^NE^ and ADV^PPE^ (Fig. 5H). Furthermore, HMGB1 knockdown reduced T_EM_/T_E_ infiltration in the tumor microenvironment (Fig. 5I). Further in vitro experiments using BMDMs and RAW264.7 cells demonstrated that the culture supernatant from HMGB1-deficient tumor cells treated with recombinant viruses failed to induce M1 polarization in BMDMs (Fig. 5J and Fig. S5A). This finding was also supported by changes in the mRNA expression levels of relevant cytokines (Fig. 5K and Fig. S5B) and chemokines (Fig. 5L and Fig. S5C), as well as alterations in the levels of secreted IL-6 and IL-10 (Fig. 5M and Fig. S5D).

In summary, we demonstrated that NE or PPE expressed by the recombinant viruses ADV^NE^ and ADV^PPE^ induces the release of HMGB1 from tumor cells, leading to the M1 polarization of macrophages in the tumor microenvironment. Together with previous results, these findings indicate that M1 polarization further promotes T_EM_/T_E_ infiltration, counteracting the negative immune feedback observed during ADV^Ctrl^ treatment.

### HMGB1 release from tumor cells treated with ADV^NE^ or ADV^PPE^ induces M1 macrophage polarization via the TLR4‒MyD88‒NFκB‒NLRP3 (ASC) pathway

**Fig. 6:**
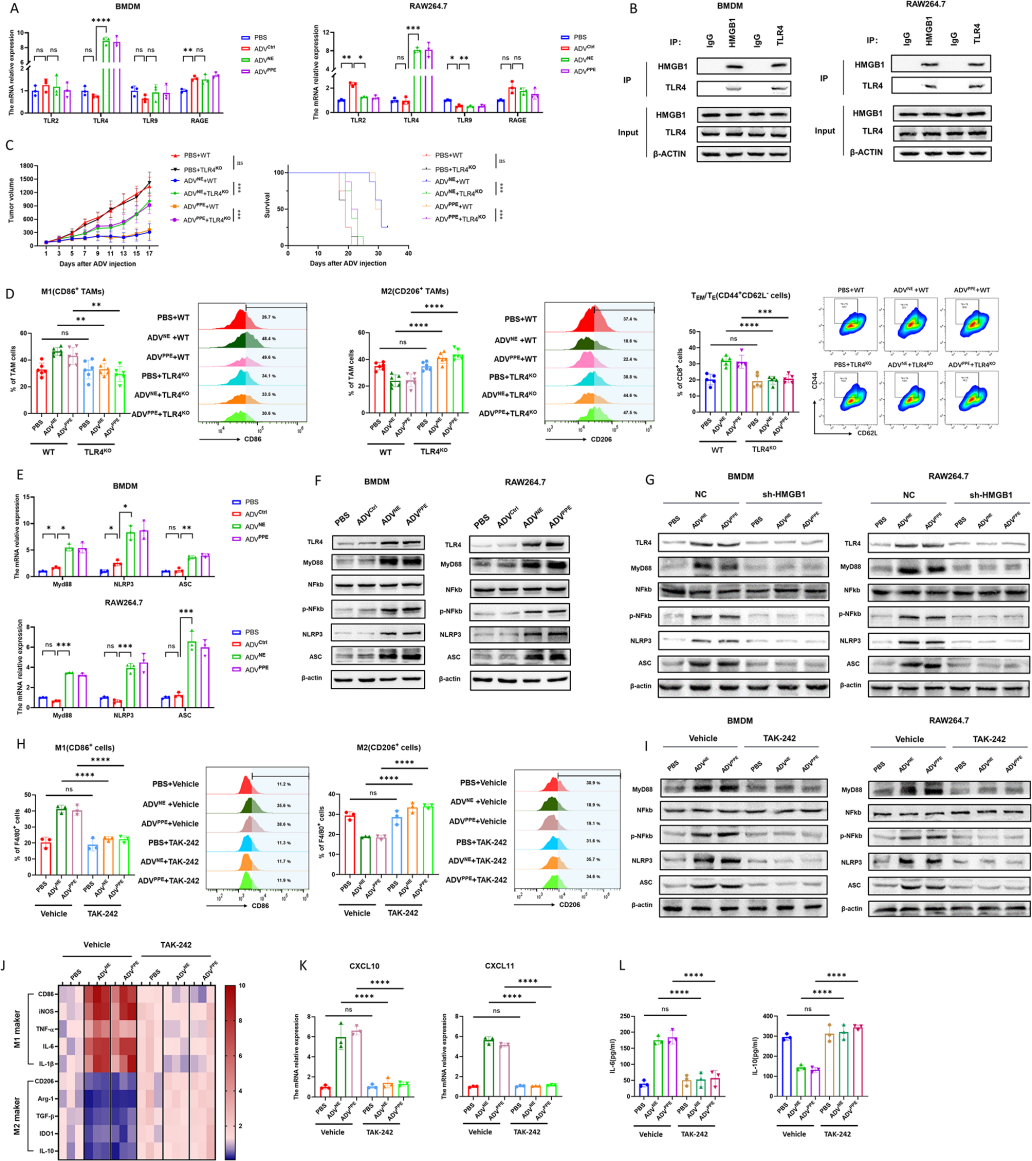
Relationship between HMGB1 released by tumor cells treated with ADV^NE^ or ADV^PPE^ and the induction of M1 macrophage polarization via the TLR4‒MyD88‒NFκB‒NLRP3 (ASC) pathway. (**A**) MC38 cells were treated with PBS, ADV^Ctrl^, ADV^NE^, or ADV^PPE^. After 24 h, the supernatant was collected to stimulate BMDMs and RAW264.7 cells. After another 24 h, qPCR was used to analyze the expression of the HMGB1-related receptors TLR2, TLR4, TLR9, and RAGE on the surfaces of BMDMs and RAW264.7 cells (*n* = 3 biological replicates). (**B**) Co-IP experiments were performed to examine the interaction between HMGB1 and the TLR4 receptor. The cell lysates were immunoprecipitated with anti-HMGB1 and anti-IgG antibodies, followed by immunoblotting with anti-HMGB1 and anti-TLR4 antibodies. Additionally, the lysates were immunoprecipitated with anti-TLR4 and anti-IgG antibodies, followed by immunoblotting with anti-HMGB1 and anti-TLR4 monoclonal antibodies. (**C**) Subcutaneous MC38 tumor models were established in TLR4 knockout mice and wild-type mice, followed by treatment with PBS, ADV^NE^, or ADV^PPE^. Subcutaneous tumor volume and survival were monitored every two days (*n* = 8 mice per group). (**D**) Following the protocol shown in **C**, flow cytometry was used on the 7th day after oncolytic virus treatment to assess M1 macrophage, M2 macrophage, and T_EM_/T_E_ infiltration in tumor tissues (*n* = 6 mice per group). (**E**) MC38 cells were treated with different viruses, and 24 h later, the supernatant was collected to stimulate BMDMs and RAW264.7 cells. After another 24 h, qPCR was used to assess MyD88, NLRP3, and ASC expression in BMDMs and RAW264.7 cells (*n* = 3 biological replicates). (**F**) BMDMs and RAW264.7 cells were stimulated with supernatants from different treatment groups for 48 h, followed by Western blot analysis to detect the protein expression levels of TLR4, MyD88, NFκB, p-NFκB, NLRP3, and ASC. (**G**) shHMGB1-MC38 and NC-MC38 cells were treated with different viruses. After 24 h, the supernatant was collected to stimulate BMDMs and RAW264.7 cells, and Western blotting was performed 48 h later to detect the expression levels of TLR4, MyD88, NFκB, p-NFκB, NLRP3, and ASC. (**H**) BMDMs were stimulated with supernatants from different treatment groups in the presence of vehicle or TAK-242. After 24 h, flow cytometry was used to assess the macrophage polarization status (*n* = 3 biological replicates). (**I**) BMDMs and RAW264.7 cells were stimulated with supernatants from different treatment groups in the presence of vehicle or TAK-242 for 48 h, followed by Western blot analysis of the proteins mentioned above. (**J**) BMDMs were stimulated with supernatants from different treatment groups in the presence of vehicle or TAK-242 for 24 h. qPCR was then used to assess M1 and M2 gene expression, and the expression levels are presented in a heatmap (*n* = 3 biological replicates). (**K**) Following the protocol shown in **J**, qPCR was used to analyze the M1 macrophage-related chemokine expression levels of CXCL10 and CXCL11 in BMDMs (*n* = 3 biological replicates). (**L**) Supernatants from different treatment groups were used to stimulate BMDMs in the presence of either the vehicle control or TAK-242. After 24 h, ELISA was performed to measure IL-6 and IL-10 levels in the BMDM culture supernatants. (*n* = 3 biological replicates). The data are presented as the means ± SDs. NS, no significant difference; ∗*p* < 0.05, ∗∗*p* < 0.01, ∗∗∗*p* < 0.001, ∗∗∗∗*p* < 0.0001

Studies have reported that HMGB1 interacts with receptors on macrophages, modulating inflammatory and immune responses. These receptors include TLR2, TLR4, TLR9, and RAGE [42–45]. Using qPCR, we examined the mRNA expression levels of these HMGB1 receptors (TLR2, TLR4, TLR9, and RAGE) in BMDMs and RAW264.7 cells. The results revealed a significant increase in TLR4 mRNA expression in the ADV^NE^ and ADV^PPE^ treatment groups compared with the ADV^Ctrl^ treatment group, with no similar changes observed for the TLR2, TLR9, or RAGE receptors (Fig. 6A). Additionally, coimmunoprecipitation (co-IP) experiments confirmed the interaction between HMGB1 and TLR4 (Fig. 6B). In MC38 subcutaneous tumor models generated with TLR4 knockout mice, we further demonstrated that the enhanced antitumor immune effects of ADV^NE^ and ADV^PPE^ depend on the presence of the TLR4 receptor (Fig. 6C). In the absence of TLR4, the previously observed increase in the proportion of M1 macrophages and T_EM_/T_E_ infiltration and the decrease in the proportion of M2 macrophages associated with ADV^NE^ and ADV^PPE^ treatments were abrogated (Fig. 6D).

Previous studies have suggested that TLR4 induces M1 macrophage polarization through MyD88 activation and NFκB phosphorylation and activates the NLRP3 inflammasome to promote proinflammatory cytokine release [46, 47]. To investigate this pathway, we treated MC38 tumor cells with PBS, ADV^Ctrl^, ADV^NE^, or ADV^PPE^, collected the culture supernatants, and used them to stimulate BMDMs and RAW264.7 cells. qPCR analysis revealed that the supernatants from recombinant virus-treated cells significantly increased the mRNA expression of MyD88, NLRP3, and ASC in macrophages (Fig. 6E). Western blotting further confirmed that the supernatants from ADV^NE−^ or ADV^PPE^-treated tumor cells activated the TLR4-MyD88-phosphorylated NFκB-NLRP3 (ASC) pathway in BMDMs (Fig. 6F). In contrast, knocking down HMGB1 in MC38 cells abolished these effects in macrophages (Fig. 6G).

TAK-242, a well-established inhibitor of TLR4, binds directly to the Cys747 residue in the intracellular domain of TLR4 to suppress its signaling [48]. Following TAK-242 treatment to inhibit TLR4 signaling, the HMGB1-induced M1 macrophage polarization stimulated with the supernatants from ADV^NE−^ or ADV^PPE^-treated tumor cells was abrogated (Fig. 6H and Fig. S6A). Additionally, MyD88-NFκB-NLRP3 (ASC) protein expression downstream of TLR4 was decreased (Fig. 6I). Changes in cytokine (Fig. 6J and Fig. S6B) and chemokine mRNA expression (Fig. 6K and Fig. S6C), along with IL-6 and IL-10 secretion levels (Fig. 6L and Fig. S6D), further corroborated these findings.

In summary, we demonstrated that HMGB1 released from tumor cells treated with the recombinant adenoviruses ADV^NE^ or ADV^PPE^ binds to the TLR4 receptor on macrophages, activating the MyD88-NFκB-NLRP3 (ASC) pathway and inducing M1 macrophage polarization.

### The recombinant advs ADV^NE^ and ADV^PPE^ show promising potential for clinical translation

**Fig. 7:**
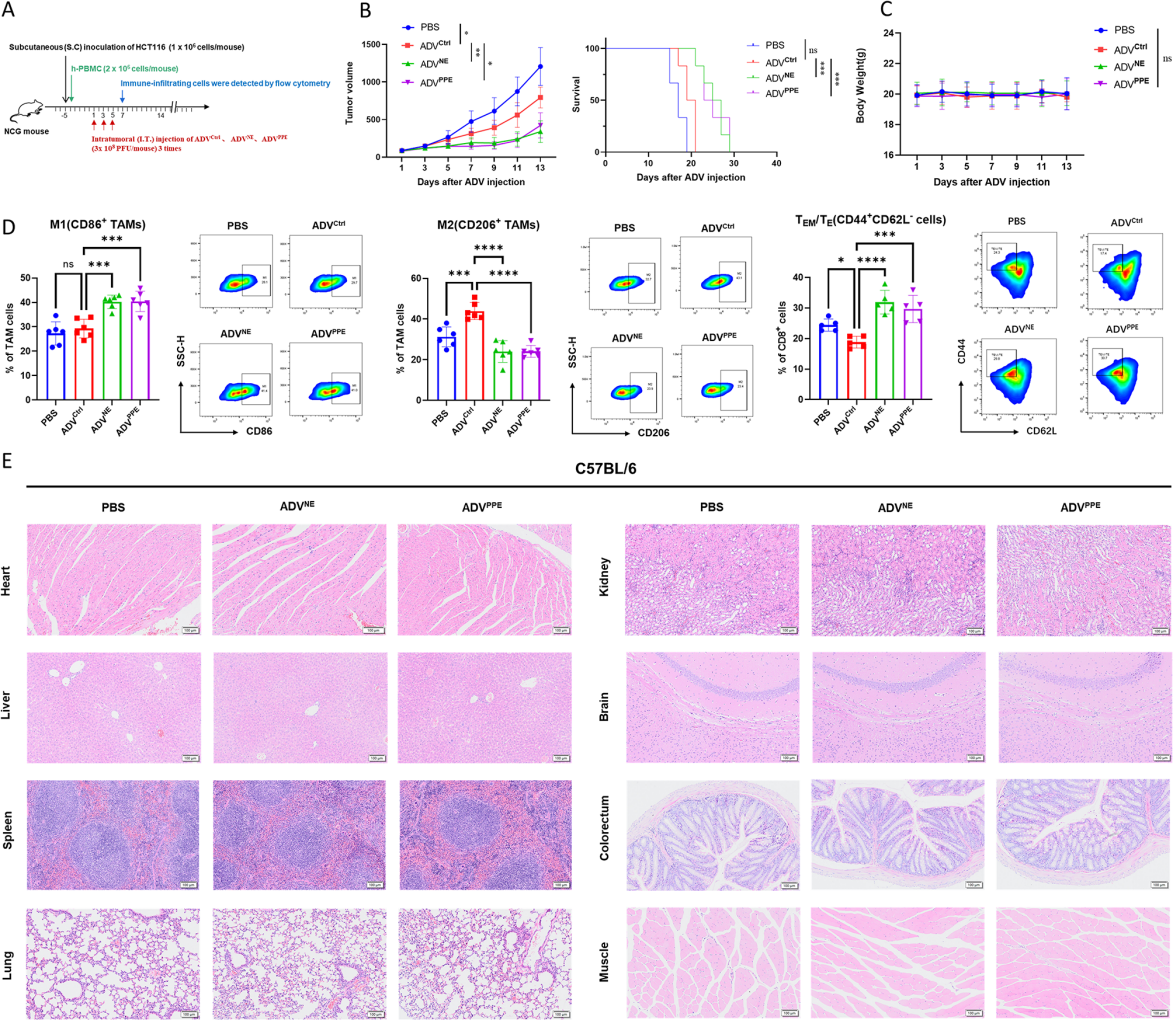
Anti-tumor immunotherapeutic effects of ADV^NE^ and ADV^PPE^ in a humanized CDX model, characterization of specific immune cells in the tumor microenvironment, and preliminary safety observations. (**A**) Schematic of the mouse treatment model for **B**-**D**. NCG mice were subcutaneously inoculated with 1 × 10⁶ HCT116 cells. On the day following tumor implantation, human peripheral blood mononuclear cells were administered via tail vein injection. When the tumors reached a volume of 50–100 mm³, intratumoral injections of 3 × 10⁸ PFU of adenovirus were administered every other day for a total of three treatments (*n* = 6 mice per group). (**B**) Changes in subcutaneous tumor volume and survival were recorded every two days following different virus treatments (*n* = 6 mice per group). (**C**) Mouse body weight was measured every two days throughout the survival observation period (*n* = 6 mice per group). (**D**) On day 7 after the first virus treatment, flow cytometry was used to assess the infiltration of M1 macrophages, M2 macrophages, and T_EM_/T_E_ cells in the tumor microenvironment (*n* = 6 mice per group). (**E**) An MC38 subcutaneous tumor model was established in C57BL/6 mice. Forty-eight hours after the third recombinant virus treatment, the heart, liver, spleen, lungs, kidneys, brain, small intestine, and muscle tissues were collected for H&E staining. The data are presented as the means ± SDs. NS, no significant difference; ∗*p* < 0.05, ∗∗*p* < 0.01, ∗∗∗*p* < 0.001, ∗∗∗∗*p* < 0.0001

To further confirm the clinical translation potential of the recombinant ADVs ADV^NE^ and ADV^PPE^, we used severely immunodeficient NCG mice to establish a humanized HCT116 subcutaneous tumor model. Following three treatment sessions, we evaluated mouse survival and immune infiltration changes in the tumor microenvironment (Fig. 7A). Compared with the ADV^Ctrl^ treatment group, the groups treated with ADV^NE^ and ADV^PPE^ presented significantly greater antitumor immunotherapy efficacy in the humanized HCT116 model (Fig. 7B), with no significant differences in body weight observed among the groups (Fig. 7C). The flow cytometry results indicated that treatment with ADV^NE^ or ADV^PPE^ increased T_EM_/T_E_ and M1 macrophage infiltration in the tumor microenvironment and reduced the proportion of M2 macrophages compared with those in the ADV^Ctrl^ group (Fig. 7D). Additionally, to assess the safety profile of the recombinant virus, we employed immunocompetent C57BL/6 mice to simulate the natural antiviral immune response under physiological conditions. H&E staining of mouse tissues revealed no obvious damage to organs from the recombinant virus treatments (Fig. 7E). The results from the humanized CDX mouse model and preliminary safety assessments suggest that ADV^NE^ and ADV^PPE^ have excellent potential for clinical translation.

## Discussion

The rapid development of molecular biotechnology has opened new avenues for the use of OVs in cancer treatment. Over the past three decades, most clinical and preclinical research has focused on OV modifications, marking the beginning of a new era in targeted cancer virotherapy with reduced toxicity [49]. Currently employed ADV modifications mainly aim to enhance tumor targeting, intratumoral spread, and the regulation of anti-ADV and antitumor immunity [50, 51]. To enhance the ability of ADV to modulate antitumor immune responses, efforts have focused primarily on remodeling the tumor immune microenvironment by using ADVs as vectors to express genes encoding costimulatory molecules, cytokines, chemokines, and tumor-associated antigens [52, 53]. However, few studies have examined the direct effects of ADVs on the tumor microenvironment itself. In this study, we observed for the first time that while ADV treatment positively impacts the tumor microenvironment, it also has the significant drawback of reducing CD8 T_eff_ infiltration. To address this, we adopted a “leveraging strengths to offset weaknesses” approach in designing a novel therapeutic oncolytic ADVs. After confirming their increased antitumor efficacy, we further investigated the underlying mechanisms contributing to this improved performance, laying a theoretical foundation for future clinical translation.

Since NE and PPE are harmless to healthy cells and cytotoxic to tumor cells, they have been used in the development of various antitumor products [54, 55]; however, they have not previously been applied in OV modification. Thus, we pioneered this approach. After the new ADVs were successfully constructed, our in vitro and in vivo experiments confirmed that ADV^NE^ and ADV^PPE^ exhibited significantly increased antitumor activity. Interestingly, our in vitro experiments revealed that the expression of NE/PPE selectively increased the oncolytic capacity of ADVs without affecting their replication. According to the findings of Cui et al., NE and PPE can induce apoptosis in tumor cells through CD95, accompanied by the accumulation of cleaved Caspase-3 [27]. In cells with high Gasdermin E (GSDME) expression, cleaved Caspase-3 can cleave GSDME, producing pore-forming GSDME-N fragments, thereby inducing pyroptosis [56, 57]. Our study confirmed this finding; compared with ADV^Ctrl^, the recombinant adenoviruses ADV^NE^ and ADV^PPE^, which express NE and PPE, indeed induce pyroptosis in tumor cells, accompanied by the release of HMGB1—an essential factor driving the changes in the tumor microenvironment observed in this study.

Macrophages and CD8^+^ T cells play pivotal roles in tumor immunotherapy, and complex interactions exist between them within the tumor microenvironment [33, 58, 59]. In this study, we observed that recombinant ADVs modified the changes in the tumor microenvironment induced by traditional ADV treatment, primarily affecting macrophage polarization and T_EM_/T_E_ infiltration. Furthermore, we confirmed that the polarization state of macrophages is a crucial factor influencing T_EM_/T_E_ infiltration. Coincidentally, a recent study supported this conclusion, showing that the use of reprogrammed M1 macrophages in antitumor therapy significantly increased T_EM_/T_E_ infiltration and achieved excellent therapeutic results [60]. Thus, decreasing the M2 polarization of macrophages and increasing the M1 polarization of macrophages are key mechanisms through which recombinant ADVs achieve increased antitumor efficacy.

There exist two primary categories of TAMs: M2 TAMs, which facilitate tumor growth, and M1 TAMs, which act to suppress tumor progression [61]. However, some scholars argue that plasticity is a hallmark of the mononuclear phagocyte system [62]. Macrophages may be more diverse than currently recognized and cannot be simply categorized into clonally distinct populations. The M1 and M2 phenotypes are more likely to represent two extreme manifestations of a phenotypic continuum [63]. Numerous factors can influence the polarization of macrophages [58, 64], and differentiation combined with polarizing signals may give rise to an even broader array of phenotypes [65]. The role of these phenotypically diverse macrophages in antitumor immunity remains an open and intriguing question worthy of further investigation. Given the crucial role of macrophages in this study, we were highly interested in understanding the mechanism by which recombinant viruses induce M1 TAM polarization. Our in vitro experiments demonstrated that the supernatant from tumor cells treated with ADV^NE^ or ADV^PPE^ promoted the M1 polarization of macrophages in the tumor microenvironment. However, this effect depends on the presence of HMGB1 in tumor cells. When HMGB1 was knocked down, the supernatant from ADV^NE−^ and ADV^PPE^-treated tumor cells lost the ability to induce the M1 polarization of macrophages. Experiments in mice further confirmed these findings. In HMGB1-deficient subcutaneous tumor model mice, both the therapeutic efficacy of ADV^NE^ and ADV^PPE^ and their ability to promote M1 macrophage polarization and T_EM_/T_E_ infiltration in the tumor microenvironment were significantly reduced. Interestingly, we observed a slight reduction in the tumor cell growth rate following HMGB1 knockdown, suggesting a protumor role of HMGB1, as reported in other studies [66–69]. Moreover, although the antitumor efficacy of ADV^NE^ and ADV^PPE^ was diminished in HMGB1-deficient tumor models, they still retained some antitumor activity, likely because of their ability to induce pyroptosis in tumor cells.

Studies have reported that the receptors involved in HMGB1-induced M1 macrophage polarization primarily include TLR2, TLR4, TLR9, and RAGE [42–45, 70]. In this study, through qPCR and coimmunoprecipitation (co-IP) experiments, we identified TLR4 as the primary receptor mediating the effects of HMGB1 during recombinant virus treatment. In vivo experiments using TLR4 knockout mice, along with in vitro studies using the TLR4 inhibitor TAK-242, further confirmed these findings. Previous research has shown that TLR4 activates macrophages through MyD88 [71], accompanied by NFκB phosphorylation [72]. NFκB activation can induce the formation of NLRP3 inflammasomes, which are among the hallmarks of M1 macrophage polarization [73, 74]. For the first time, we integrated these findings and demonstrated that during recombinant virus infection, HMGB1 binds to TLR4 on macrophages, activating the MyD88-NFκB-NLRP3 (ASC) pathway and inducing M1 polarization. Furthermore, we confirmed that either HMGB1 knockdown in tumor cells or the use of TAK-242 can inhibit this process, underscoring the critical roles of HMGB1 and the TLR4 receptor in the M1 polarization of macrophages.

**Fig. 8:**
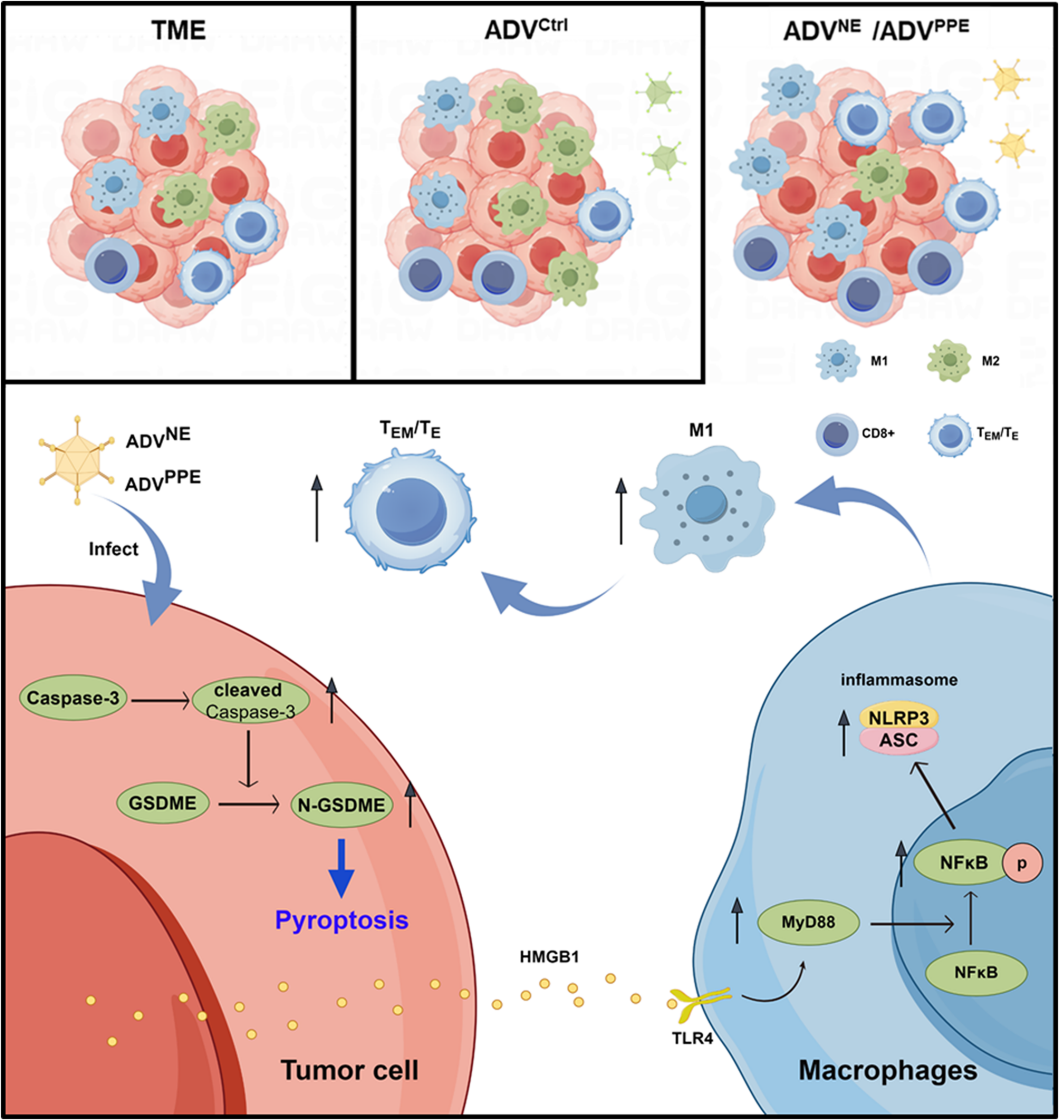
Schematic illustrating the mechanism by which ADV^NE^ and ADV^PPE^ exert enhanced therapeutic effects and abrogate negative immune feedback associated with ADV therapy. During ADV therapy, immune negative feedback manifests as reduced T_EM_/T_E_ infiltration and increased M2 macrophage infiltration. ADV^NE^ and ADV^PPE^ induce pyroptosis in colorectal cancer cells by stimulating the release of HMGB1. HMGB1 binds to TLR4 on macrophage surfaces, activating the MyD88-NFκB-NLRP3 (ASC) pathway, promoting the M1 polarization of TAMs, and subsequently increasing T_EM_/T_E_ infiltration. This process abrogates the negative immune feedback generated during ADV therapy, ultimately resulting in a robust antitumor immune response

In summary, because ADV treatment reduces T_EM_/T_E_ infiltration in the tumor microenvironment, we designed novel recombinant OVs, ADV^NE^ and ADV^PPE^, with increased antitumor efficacy. We demonstrated their ability to promote the M1 polarization of TAMs and increase T_EM_/T_E_ infiltration, explored the specific mechanisms through which they induce the M1 polarization of TAMs, and further confirmed their potential for clinical translation (Fig. 8). This study also validates the value of the “leveraging strengths to offset weaknesses” concept in antitumor therapy, presenting an effective strategy to increase the efficacy of oncolytic virotherapy. This methodology could potentially provide novel perspectives for the advancement of cancer immunotherapy.

## Limitations

This study has several limitations. First, we did not have access to samples from patients treated with ADVs, which could have provided stronger evidence confirming the observed limitation of insufficient T_EM_/T_E_ infiltration during ADV treatment. Second, although we identified the impact of macrophage polarization on T_EM_/T_E_ infiltration, the specific mechanisms underlying their interaction remain unclear and will be the focus of our future research.

## Materials and methods

### Cell lines

Human embryonic kidney cells (HEK-293T) and murine colorectal cancer cells (CT26) were obtained from the American Type Culture Collection (ATCC, USA). The murine colorectal cancer cell line MC38 was acquired from the National Cancer Institute (NCI, USA). The murine macrophage-like cell line RAW264.7 was sourced from the China Center for Type Culture Collection (CCTCC, China). All cell lines were cultured in DMEM supplemented with 10% FBS and maintained in a humidified incubator at 37 °C with 5% CO₂.

### Isolation of bone marrow-derived macrophages (BMDMs)

Bone marrow was flushed from the femurs and tibias of eight- to ten-week-old C57BL/6 mice. The bone marrow cells were filtered through a 70 μm cell strainer and resuspended at room temperature in red blood cell lysis buffer (Beyotime, China) for 5 min to remove erythrocytes. The remaining cells were then cultured at a density of 10^6^ cells/mL in complete RPMI supplemented with 20 ng/ml recombinant murine (rm) M-CSF (Invitrogen, UK) for 7 days.

### Mouse tumor models

C57BL/6 and BALB/c mice, aged 4–8 weeks, were obtained from the Nanjing University Model Animal Research Center, while NCG mice (NOD/ShiLtJGpt-Prkdc^em26Cd52^Il2rg^em26Cd22^/Gpt) were purchased from GemPharmatech. All animal experiments followed protocols approved by the Institutional Animal Care and Use Committee of Nanjing University Medical School.For the subcutaneous (S.C.) tumor model, exponentially growing MC38 or CT26 cells were collected and subcutaneously injected into the right flank of each mouse. Once tumor volumes reached 50–100 mm³, mice received intratumoral injections of PBS or different viruses every other day for a total of three treatments. Tumor growth was measured every other day, with tumor volume calculated as 0.5 × length × width². Mice were sacrificed when tumor volume reached a maximum of 1500 mm³.For the PBMC-humanized CDX model, exponentially growing HCT116 cells were injected subcutaneously into the right flank of each mouse. On the day following tumor implantation, PBMCs (obtained from human peripheral blood) were administered via tail vein injection. Treatment commenced when tumors reached a size of 50–100 mm³, following the same protocol as described above.

### In vivo immune cell depletion

For macrophage and CD8^+^ T cell depletion, C57BL/6 mice received intraperitoneal injections of 500 µg anti-CD8α (BioXCell, USA) or anti-CSF1R (BioXCell, USA) every other day for a total of three injections. One week after the final injection, flow cytometry was performed to assess the efficiency of immune cell depletion using anti-CD8α or anti-CSF1R.

### Preparation of recombinant adenovirus

Target sequences for viral genes E1A, ELANE, and CELA1 were fully synthesized by GeneScript and subcloned into the pShuttle (pENTER/D-TOPO) plasmid. To facilitate detection of the target proteins, a His-tag (HHHHHH) was added downstream of the ELANE and CELA1 sequences. The shuttle plasmid was then recombined with the adenoviral backbone vector pAd/PL-DEST (Thermo Fisher Scientific, USA) to generate recombinant adenoviral vectors expressing the target proteins. After linearization with the PacI restriction enzyme, the constructs were transfected into 293T cells to produce recombinant adenovirus. The virus was subsequently amplified in 293T cells, purified by sucrose gradient ultracentrifugation, and titrated using the TCID₅₀ assay to determine viral titers.

### CCK8 assay

CT26 and MC38 cells were seeded at a density of 1 × 10^4^ cells per well in 96-well plates and infected with various adenoviruses at specified MOIs. After 48 h of incubation, 10 µL of CCK-8 solution (Beyotime, China) was added to each well and incubated for an additional hour. Absorbance was measured at 450 nm using a microplate reader. Cell viability was calculated using the following formula: Cell Viability (%) = [(A_treatment_ − A_blank_) / (A_control_ − A_blank_)] × 100%.

### Crystal violet staining

Tumor cells were plated and infected with adenovirus following a protocol similar to that used in the CCK8 assay. After 48 h of incubation, the culture medium was removed, and crystal violet solution (Beyotime, China) was added to each well for a 5-minute incubation. Following incubation, the crystal violet solution was removed, and the wells were washed five times with ddH₂O. Images were captured using a scanner.

### Viral replication

In vitro, cells were seeded at 5 × 10^4^ cells per well in 24-well plates and allowed to reach 90% confluence. The cells were then infected with adenovirus at an MOI of 0.1. Cells were harvested at 12, 24, 48, 72, and 96 h post-infection and subjected to three freeze-thaw cycles. The viral supernatant was collected, and viral titers were determined using the TCID₅₀ assay.In vivo, blood and tumor tissues were collected from mice on the seventh day following the initial viral treatment. Tumor samples (100 mg) were homogenized in 200 µL PBS. The homogenate was centrifuged to separate the supernatant and serum. Quantitative PCR (q-PCR) was used to detect the expression of the adenovirus-specific E1A gene, enabling assessment of viral distribution.

### Elisa

Supernatants from BMDMs or tumor cells treated with various interventions were collected (1000 × g, 20 min, 4 °C). Mouse IL-6, IL-10, and HMGB1 concentrations were measured using ELISA kits (Elabscience, China) according to the manufacturer’s instructions.

### LDH release assays

Tumor cells were infected with various adenoviruses, and after 24 h, LDH release was measured using the LDH Release Assay Kit (Beyotime, China) according to the manufacturer’s instructions.

### qRT-PCR

Total RNA was extracted from cells using TRIZOL (Vazyme, China) and subsequently reverse transcribed into cDNA with the HiScript III RT SuperMix for qPCR (Vazyme, China). mRNA expression levels were analyzed on an Applied QuantStudio™ 5 Real-Time PCR System (Thermo Fisher Scientific, USA) using ChamQ SYBR qPCR Master Mix (Vazyme, China), with GAPDH serving as the internal control for normalization. Primer sequences are provided in Table 1.

**Table 1:** Primers used for qRT-PCR

Target Name	primer		
CD86-F	TGTTTCCGTGGAGACGCAAG		
CD86-R	TTGAGCCTTTGTAAATGGGCA		
iNOS-F	GTTCTCAGCCCAACAATACAAGA		
iNOS-R	GTGGACGGGTCGATGTCAC		
TNF-α-F	CCCTCACACTCAGATCATCTTCT		
TNF-α-R	GCTACGACGTGGGCTACAG		
IL-6-F	TAGTCCTTCCTACCCCAATTTCC		
IL-6-R	TTGGTCCTTAGCCACTCCTTC		
IL-1β-F	TCTTTGAAGTTGACGGACCC		
IL-1β-R	TGAGTGATACTGCCTGCCTG		
Arg-1-F	CTCCAAGCCAAAGTCCTTAGAG		
Arg-1-R	AGGAGCTGTCATTAGGGACATC		
CD206-F	CTCTGTTCAGCTATTGGACGC		
CD206-R	CGGAATTTCTGGGATTCAGCTTC		
TGF-β-F	CTCCCGTGGCTTCTAGTGC		
TGF-β-R	GCCTTAGTTTGGACAGGATCTG		
IL-12-F	CCCAGCACTGCATAAACTAAGTATG		
IL-12-R	ATTCCAAAAGCTTCTGTTCTTCCAG		
IL-10-F	GCTCTTACTGACTGGCATGAG		
IL-10-R	CGCAGCTCTAGGAGCATGTG		
IDO1-F	GCTTTGCTCTACCACATCCAC		
IDO1-R	CAGGCGCTGTAACCTGTGT		
CXCL10-F	CCAAGTGCTGCCGTCATTTTC		
CXCL10-R	GGCTCGCAGGGATGATTTCAA		
CXCL11-F	GGCTTCCTTATGTTCAAACAGGG		
CXCL11-R	GCCGTTACTCGGGTAAATTACA		
TLR2-F	CTCTTCAGCAAACGCTGTTCT		
TLR2-R	GGCGTCTCCCTCTATTGTATTG		
TLR4-F	GCCTTTCAGGGAATTAAGCTCC		
TLR4-R	GATCAACCGATGGACGTGTAAA		
TLR9-F	ATGGTTCTCCGTCGAAGGACT		
TLR9-R	CAGGTGGTGGATACGGTTGG		
Rage-F	GCCACTGGAATTGTCGATGAGG		
Rage-R	GCTGTGAGTTCAGAGGCAGGAT		
Myd88-F	TCATGTTCTCCATACCCTTGGT		
Myd88-R	AAACTGCGAGTGGGGTCAG		
NLRP3-F	ATTTGTACCCAAGGCTGCTA		
NLRP3-R	GCGGGTAATCTTCCAAATGC		
ASC-F	AGTCTGGAGCTGTGGCTACTGC		
ASC-R	TGAGTGCTTGCCTGTGTTGGTC		
Hmgb1-F	GCTGACAAGGCTCGTTATGAA		
Hmgb1-R	CCTTTGATTTTGGGGCGGTA		

### Lentiviral transfection

Following the manufacturer’s instructions, MC38 cells were transfected with either control lentivirus (pLKO.1 or pCMV) or lentivirus containing an HMGB1 knockdown construct (Genepharma, China). Stable transfectants were selected using puromycin hydrochloride (MedChemExpress, China). Knockdown efficiency was confirmed by RT-PCR and Western blot analysis. Details regarding the shRNA sequences used can be found in Supplementary Table 1.

### Co-immunoprecipitation (CO-IP) and western blot

For CO-IP, cells were lysed using Pierce IP lysis buffer (Thermo Fisher Scientific, USA) containing protease inhibitor cocktail (MedChemExpress, China). Cell lysates were incubated overnight at 4 °C with anti-HMGB1 antibody (ab182561, 1:200, Abcam) or control IgG (40 µL Protein A/G PLUS-Agarose, No. 17061801, GE Healthcare), as well as with anti-TLR4 antibody (ab182561, 1:200, Abcam) or control IgG. Beads were washed three times with PBS before proceeding to immunoblot analysis. For Western blot, cells were lysed using RIPA buffer (Beyotime, China) containing protease and phosphatase inhibitors (NCM Biotech, China). Protein concentrations were determined using the Enhanced BCA Protein Assay Kit (Beyotime, China). Proteins were separated by SDS-PAGE and transferred onto PVDF membranes (Thermo Fisher Scientific, USA). Membranes were blocked with 5% non-fat milk, incubated overnight at 4 °C with primary antibodies, followed by incubation with horseradish peroxidase-conjugated anti-rabbit or anti-mouse IgG secondary antibodies. Blot images were captured using a chemiluminescent detection system (BIO-RAD, ChemiDoc™ MP Imaging System). Primary antibodies used were anti-HMGB1 (ab18256, 1:1000, Abcam), anti- Gasdermin E (88874 S, 1:1000, CST), anti-Caspase-3 (9662 S, 1:1000, CST), anti-TLR4 (sc-293072, 1:1000, Santa), anti-MyD88 (sc-74532, 1:1000, Santa), anti-NF-κB p65 (8242 S, 1:1000, CST), anti-p-NF-κB p65 (3033 S, 1:1000, CST), anti-NLRP3 (19771–1-AP, 1:1000, Proteintech), anti-ASC (sc-514414, 1:1000, Santa) and anti-β- Actin (66009-1-Ig,1: 20000, Proteintech).

### Flow cytometry

For apoptosis analysis, tumor cells were infected with various adenoviruses at an MOI of 20. After 24 h, cells were collected and stained with Annexin-V/7AAD. For macrophage polarization assays in vitro, cells were harvested at specified time points following different treatments and stained with respective antibodies. In vivo, extracted tumors were cut into small fragments and incubated at 37 °C for 1 h in a mixture containing DMEM and type IV collagenase (50 µg/mL). The tumor tissue was then filtered to create a single-cell suspension and stained with specific antibodies. To exclude dead cells, 4′,6-diamidino-2-phenylindole (DAPI) were added shortly before analysis or sorting. Samples were processed on a Beckman Coulter Cytoflex S and analyzed using FlowJo 10 software. Fluorescent antibodies recognizing murine CD45-APC, CD4-PE/Cyanine7, CD11b-FITC, CD49b-PE, NK1.1-FITC, F4/80-PE/ Cyanine7, CD86-PE, CD206-PerCP/Cy5.5, CD3-APC/Cyanine7, CD8-PerCP/Cy5.5, CD62L-APC, CD44-PE, CD45-FITC, CD11c-PE/ Cyanine7, and IA/IE- APC/Cyanine7 were used in this assay and acquired from BioLegend.

### Hematoxylin and eosin (H&E) staining

Mouse tissues were fixed in 4% (w/v) paraformaldehyde (Sigma-Aldrich, Germany) and embedded in paraffin blocks. Sections of 5 μm thickness were cut from the paraffin-embedded blocks, deparaffinized in xylene, and rehydrated through a graded ethanol series. Standard H&E staining was then performed.

### Statistical analysis

All statistical analyses were performed using Prism 9 (GraphPad Software Inc., CA, USA). Data are presented as mean ± standard deviation (SD). Differences were analyzed using t-tests or analysis of variance (ANOVA) as appropriate. Survival curves were generated using the Kaplan-Meier method, and statistical significance was assessed using the log-rank test. Statistical significance was defined as P-values < 0.05, with thresholds indicated as *p < 0.05, **p < 0.01, ***p < 0.001, and ****p < 0.0001.

## Electronic supplementary material

Below is the link to the electronic supplementary material.

Supplementary Material 1

## Data Availability

No datasets were generated or analysed during the current study.
