# Supplementary material for: Viral expression of NE/PPE enhances anti-colorectal cancer efficacy of oncolytic adenovirus by promoting TAM M1 polarization to reverse insufficient effector memory/effector CD8+ T cell infiltration
Source: J Exp Clin Cancer Res. 2025 Mar 14;44:97. doi: 10.1186/s13046-025-03358-y (PMC11907943; doi:10.1186/s13046-025-03358-y)
Supplement: Supplementary file 1 — Supplementary Material 1 [file 13046_2025_3358_MOESM1_ESM.docx]

**
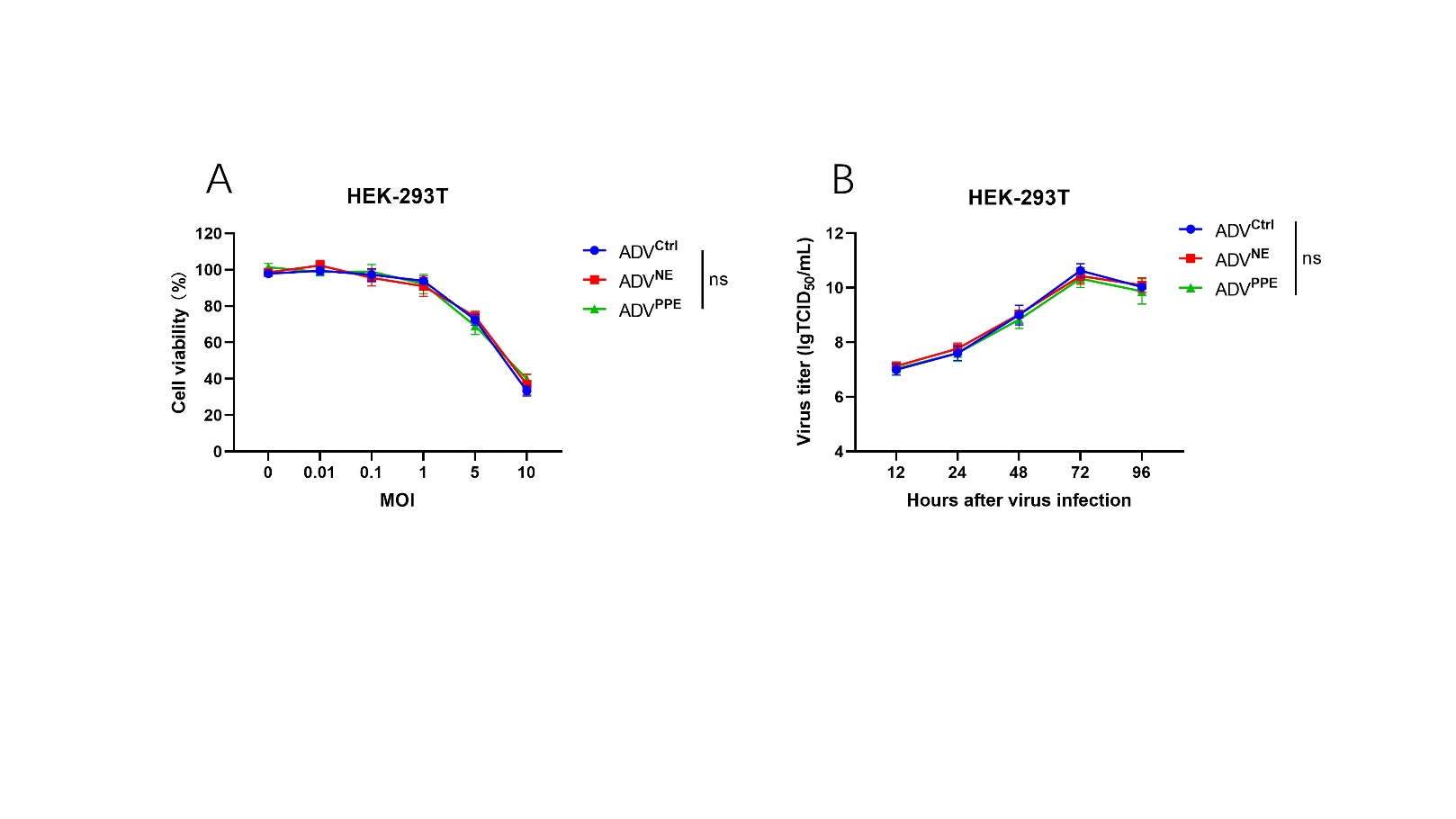
**

**Figure S1.** The effects of ADV^Ctrl^ and recombinant virus on HEK-293T cells. Related to Figure 2.

(A) To evaluate the oncolytic activity of the recombinant ADVs, HEK-293T cells were infected with ADV^Ctrl^, ADV^NE^, or ADV^PPE^ at different multiplicities of infection (MOIs). Cytotoxicity was assessed 48 hours post infection via a CCK-8 assay (n = 3 biological replicates).

(B) To assess the replication capacity of oncolytic ADVs, HEK-293T cells were infected at an MOI of 0.1, and viral titers were measured at 12 h, 24 h, 48 h, 72 h, and 96 h post infection via a TCID_50_ assay (n = 3 biological replicates).

The data are presented as the means ± SDs. NS, no significant difference; ∗p < 0.05, ∗∗p < 0.01, ∗∗∗p < 0.001, ∗∗∗∗p < 0.0001.


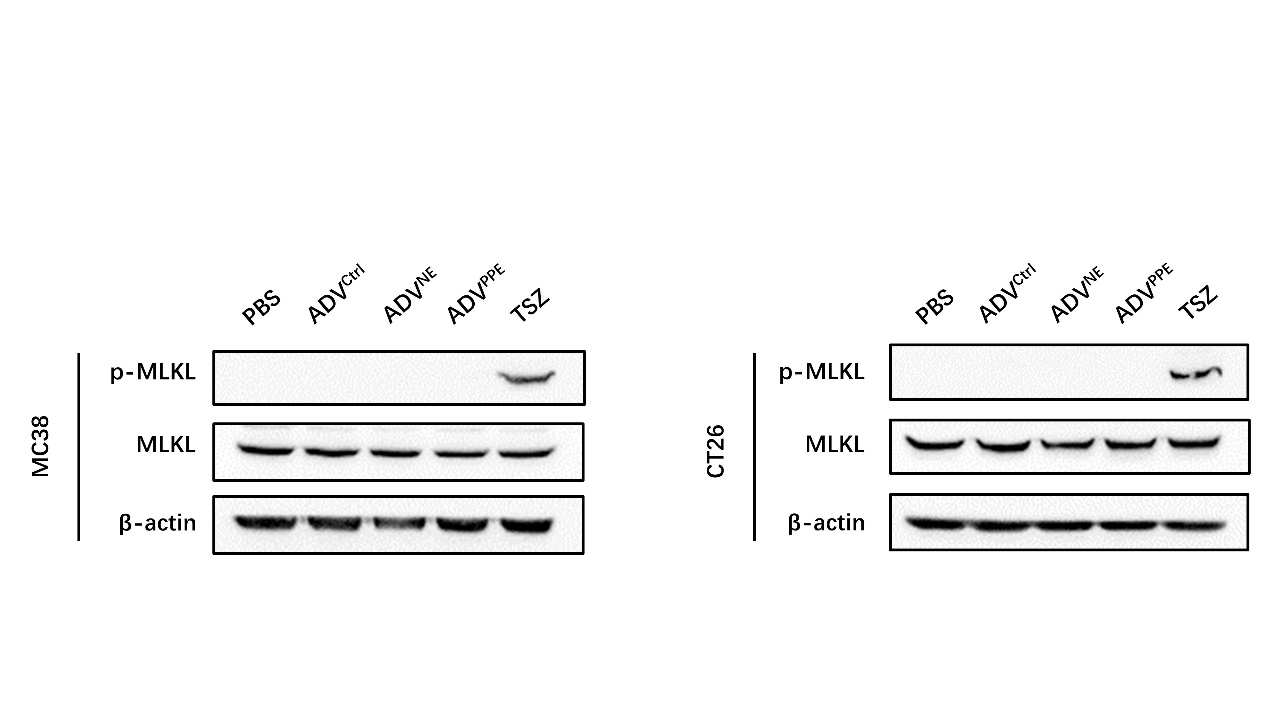


**Figure S2.** The impact of recombinant oncolytic adenovirus on the induction of necroptosis in tumor cells

After infecting MC38 and CT26 cells with different viruses, the intracellular levels of MLKL and phosphorylated MLKL (p-MLKL) were detected using Western blot. The Necroptosis Inducer Kit with TSZ was used to induce necroptosis in tumor cells as a positive control.


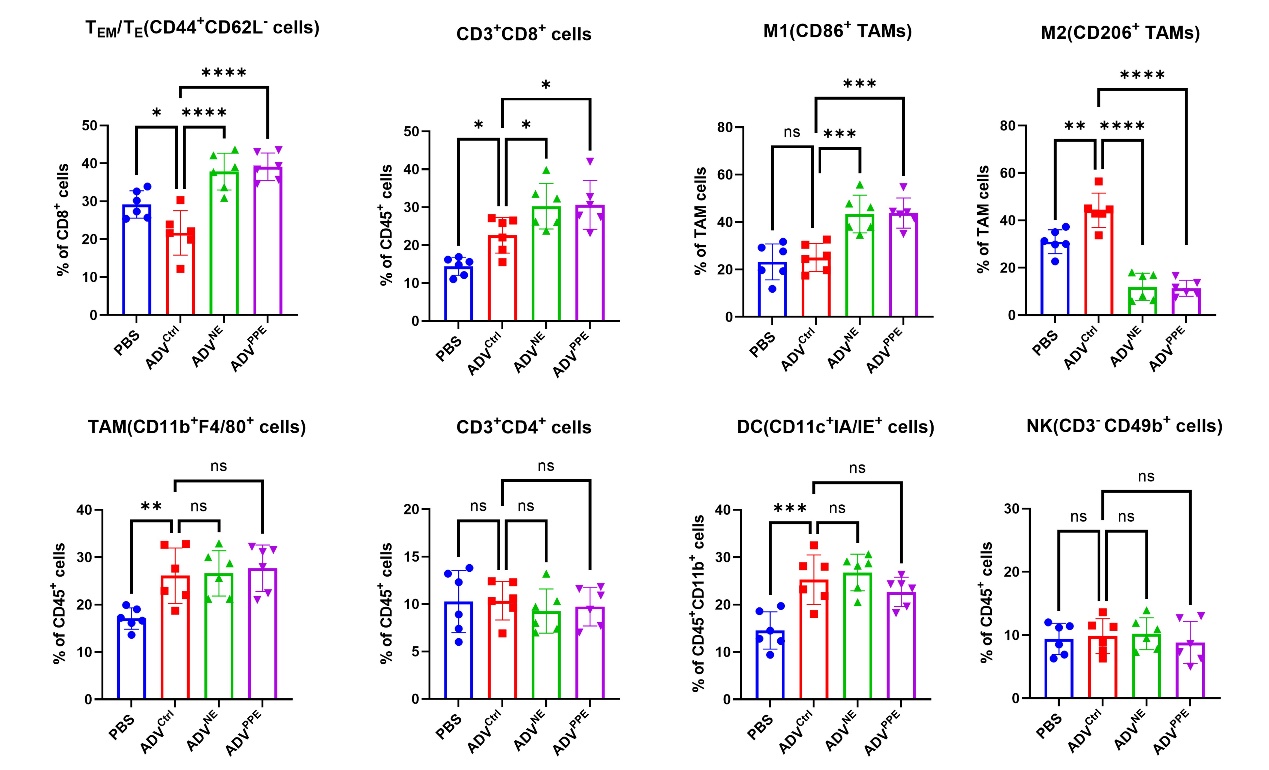


**Figure S3.** The effects of different oncolytic adenovirus therapies on the tumor microenvironment (TME) in a mouse CT26 subcutaneous tumor model. Related to Figure 4.

BALB/c mice were subcutaneously inoculated with 1x10⁶ CT26 cells. When the tumors reached a volume of 50–100 mm³, intratumoral injections of 3×10⁸ PFU of ADV were administered every other day for a total of three treatments. On day 7 after the first virus treatment, flow cytometry was used to assess the proportions of CD8^+^ T_eff_s, CD8^+^ T cells, M1 macrophages, M2 macrophages, TAMs, CD4^+^ T cells, DCs and NK cells in the tumor microenvironment of mice following different virus treatments (n = 6 mice per group).

The data are presented as the means ± SDs. NS, no significant difference; ∗p < 0.05, ∗∗p < 0.01, ∗∗∗p < 0.001, ∗∗∗∗p < 0.0001.


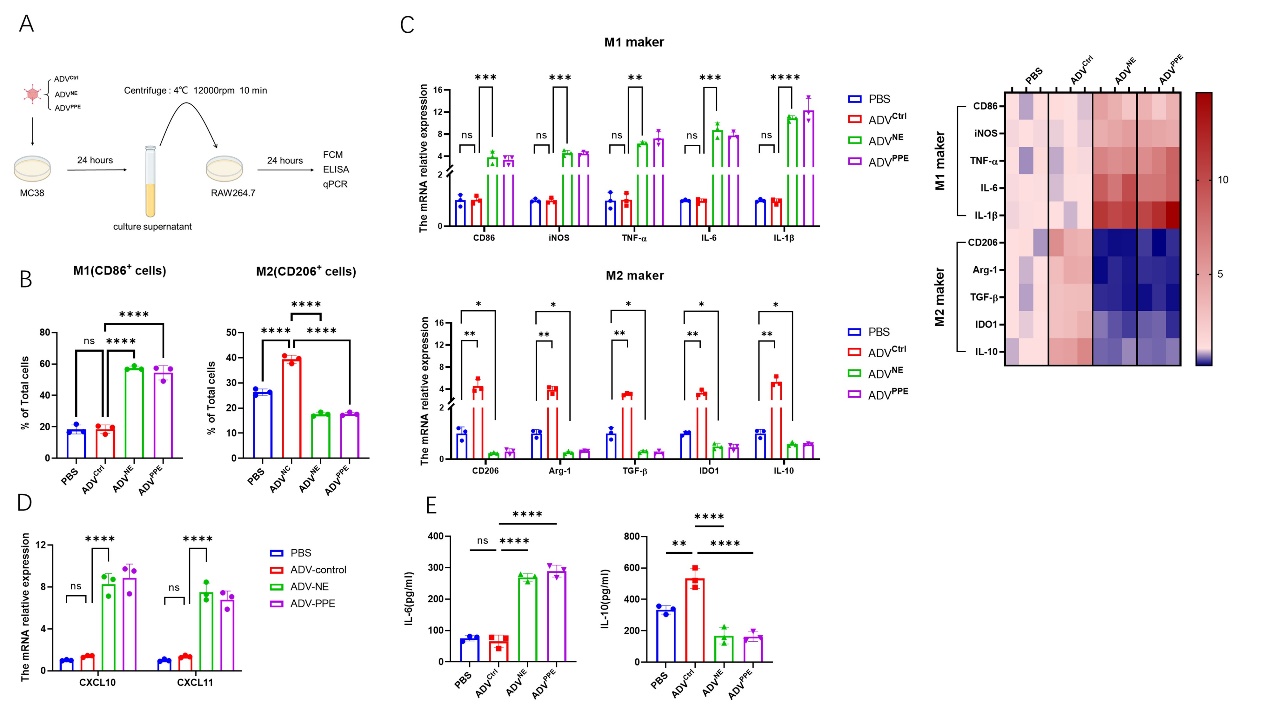


**Figure S4.** Effects of tumor cell supernatants from different oncolytic adenovirus treatment groups on M1 polarization of RAW264.7 cells. Related to Figure 5.

(A) Schematic of the in vitro experimental model shown in S3B-S3E: MC38 cells were treated with PBS, ADV^Ctrl^, ADV^NE^, or ADV^PPE^, and after 24 hours, the tumor cell supernatant was collected to stimulate RAW264.7 cells. Flow cytometry, qPCR, and ELISA were performed 24 hours after stimulation.

(B) Polarization state of RAW264.7 cells after 24 hours of stimulation with supernatants from different treatment groups, as assessed by flow cytometry (n = 3 biological replicates).

(C) qPCR analysis of gene expression in RAW264.7 cells for CD86, iNOS, TNF-α, IL-6, IL-1β, Arg-1, CD206, TGF-β, IDO1, and IL-10, with expression levels presented as a heatmap (n = 3 biological replicates).

(D) qPCR analysis of M1 macrophage-related chemokines CXCL10 and CXCL11 in RAW264.7 cells (n = 3 biological replicates).

(E) ELISA of IL-6 and IL-10 levels in the supernatants of RAW264.7 cells after 24 hours of stimulation with different treatments (n = 3 biological replicates).

The data are presented as the means ± SDs. NS, no significant difference; ∗p < 0.05, ∗∗p < 0.01, ∗∗∗p < 0.001, ∗∗∗∗p < 0.0001.


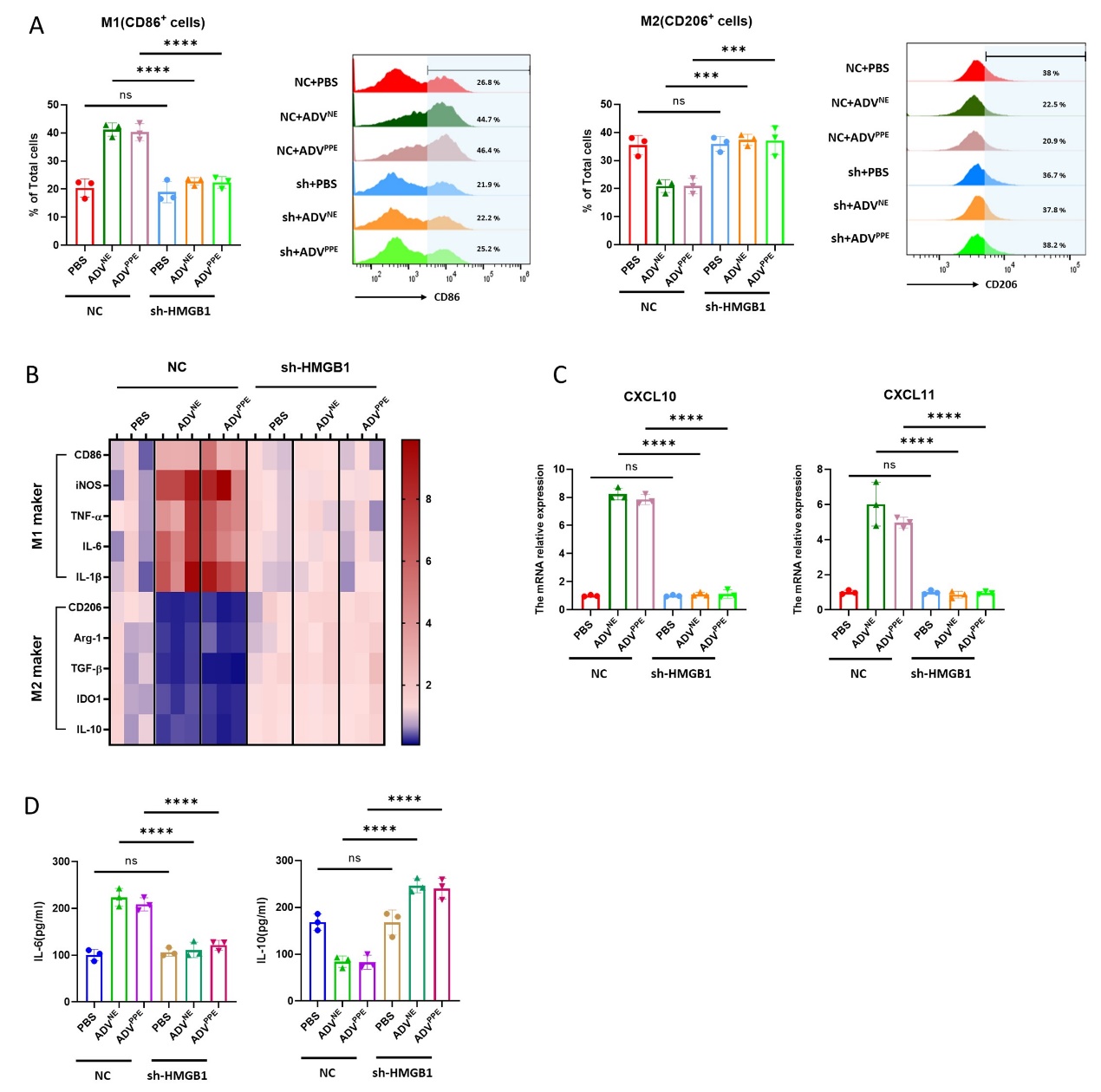


**Figure S5**. The significance of HMGB1 in promoting M1 polarization of RAW cells induced by recombinant viruses. Related to Figure 5.

(A) MC38 cells (shHMGB1-MC38 and NC-MC38) were treated with PBS, ADV^NE^, or ADV^PPE^, and after 24 hours, the tumor cell supernatant was collected to stimulate RAW264.7 cells. After an additional 24 h, the polarization of the RAW264.7 cells was assessed via flow cytometry (n = 3 biological replicates).

(B) RAW264.7 cells were stimulated with supernatants from the various treatment groups for 24 hours, qPCR was then used to assess M1 and M2 gene expression, and the expression levels are presented in a heatmap (n = 3 biological replicates)..

(C) Following the protocol shown in Figure S4B, qPCR was used to analyze the M1 macrophage-related chemokine expression levels of CXCL10 and CXCL11 in RAW264.7 cells (n = 3 biological replicates).

(D) RAW264.7 cells were stimulated with supernatants from the various treatment groups for 24 hours, ELISA was then performed to measure IL-6 and IL-10 levels in the RAW264.7 cells culture supernatants. (n = 3 biological replicates).

The data are presented as the means ± SDs. NS, no significant difference; ∗p < 0.05, ∗∗p < 0.01, ∗∗∗p < 0.001, ∗∗∗∗p < 0.0001.


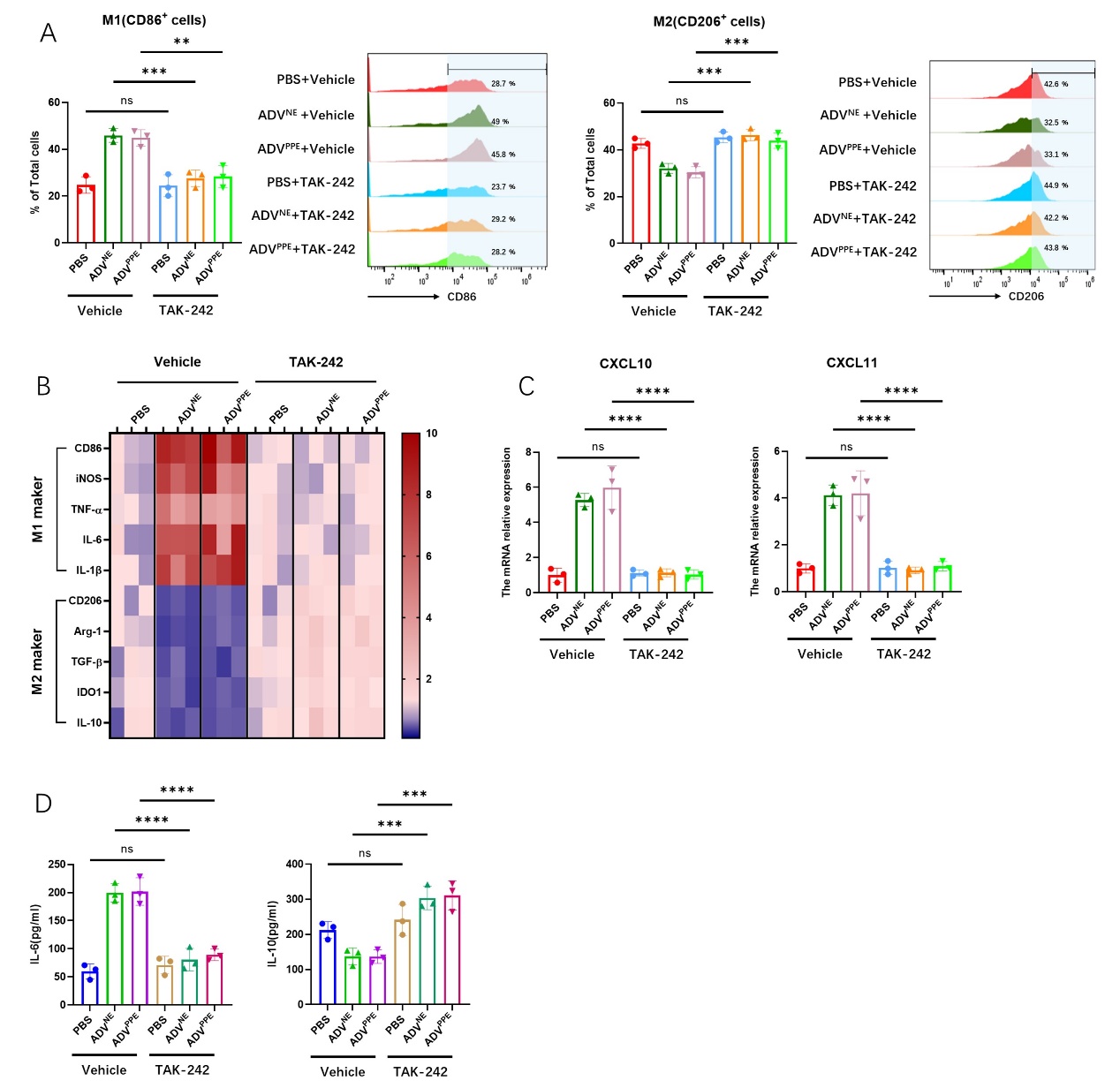


**Figure S6.** The significance of TLR4 in promoting M1 polarization of RAW cells induced by recombinant viruses. Related to Figure 6.

(A) RAW264.7 cells were stimulated with supernatants from different treatment groups in the presence of vehicle or TAK-242. After 24 hours, flow cytometry was used to assess the macrophage polarization status (n = 3 biological replicates).

(B) RAW264.7 cells were stimulated with supernatants from different treatment groups in the presence of vehicle or TAK-242 for 24 hours. qPCR was then used to assess M1 and M2 gene expression, and the expression levels are presented in a heatmap (n = 3 biological replicates).

(C) Following the protocol shown in Figure 6J, qPCR was used to analyze the M1 macrophage-related chemokine expression levels of CXCL10 and CXCL11 in RAW264.7 cells (n = 3 biological replicates).

(D) Supernatants from different treatment groups were used to stimulate RAW264.7 cells in the presence of either the vehicle control or TAK-242. After 24 hours, ELISA was performed to measure IL-6 and IL-10 levels in the BMDM culture supernatants. (n = 3 biological replicates).

The data are presented as the means ± SDs. NS, no significant difference; ∗p < 0.05, ∗∗p < 0.01, ∗∗∗p < 0.001, ∗∗∗∗p < 0.0001.


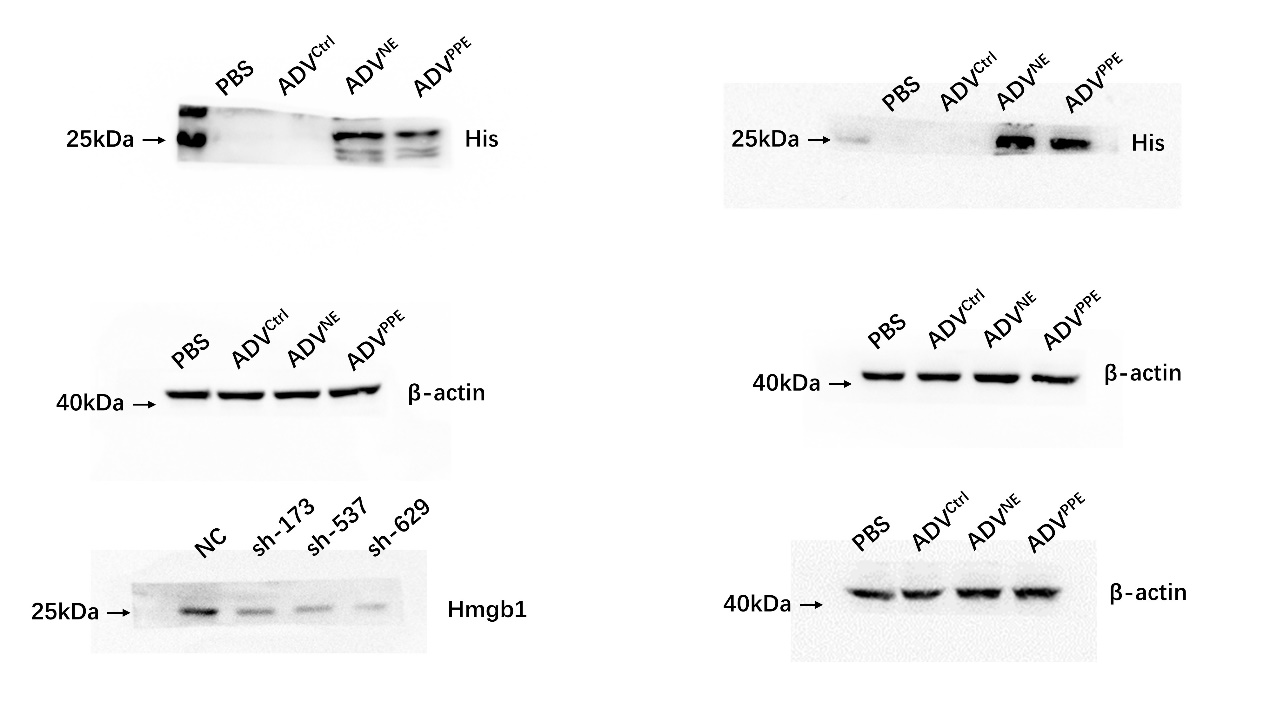


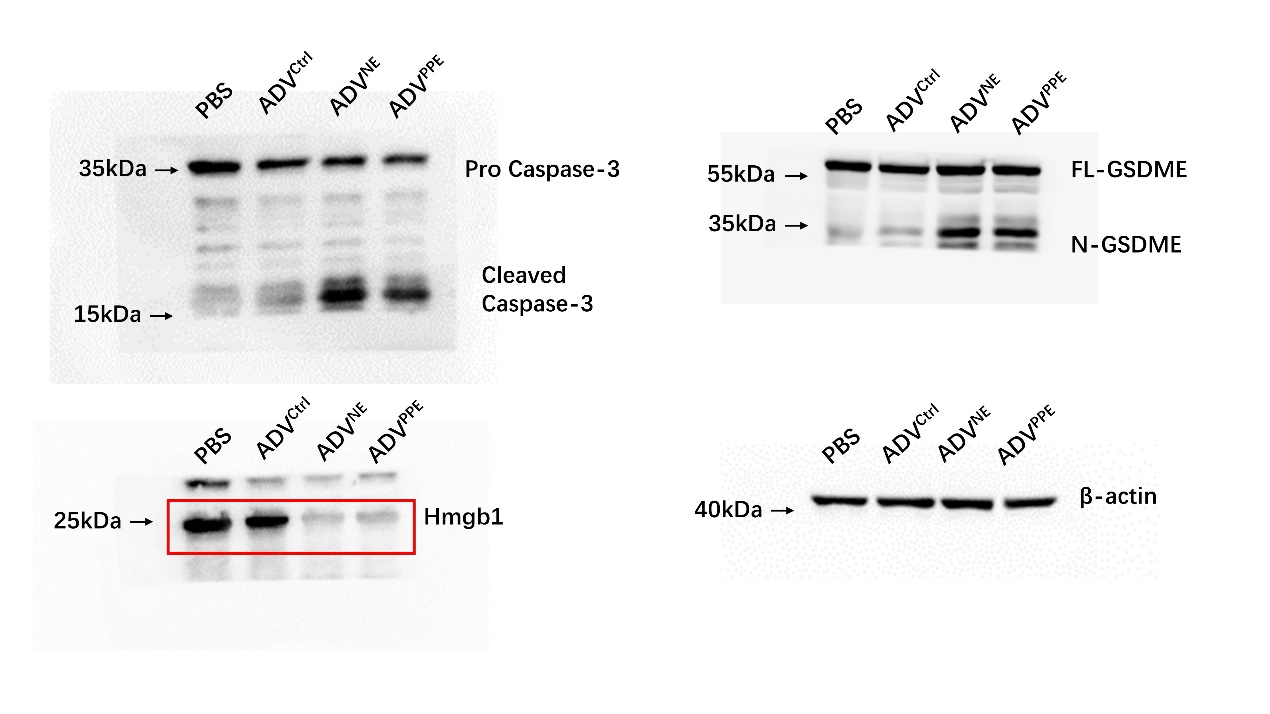

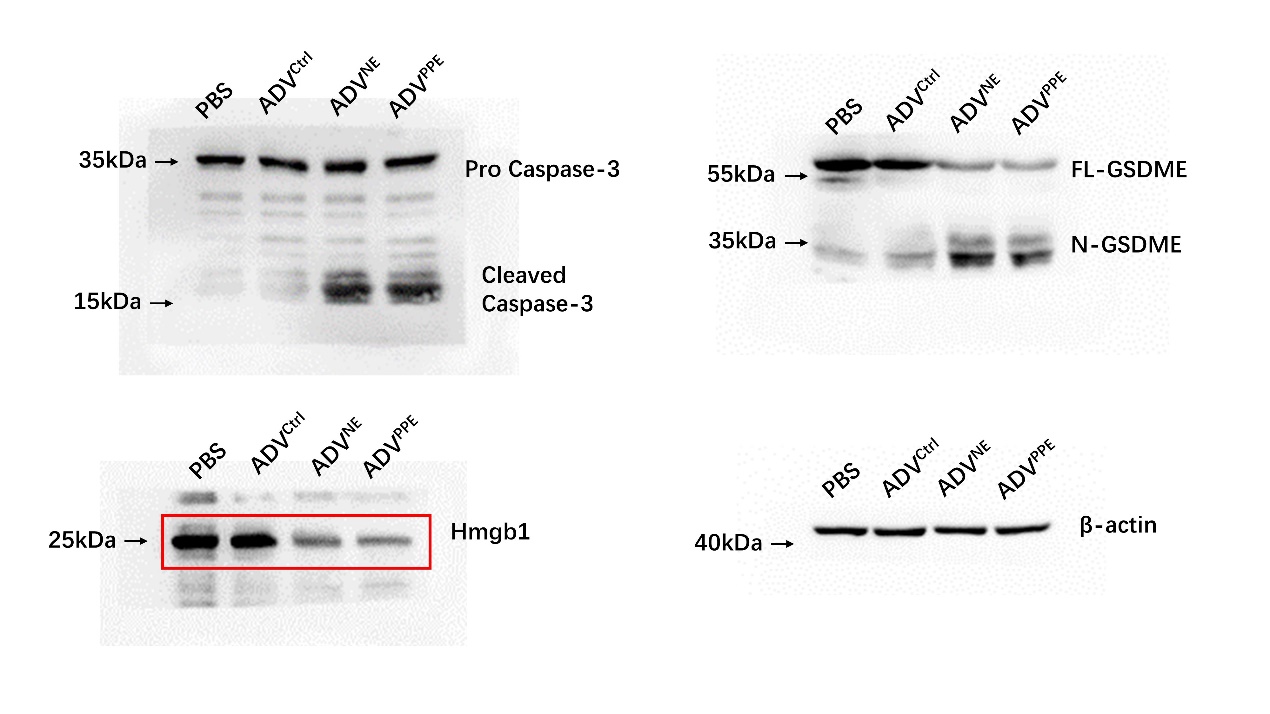


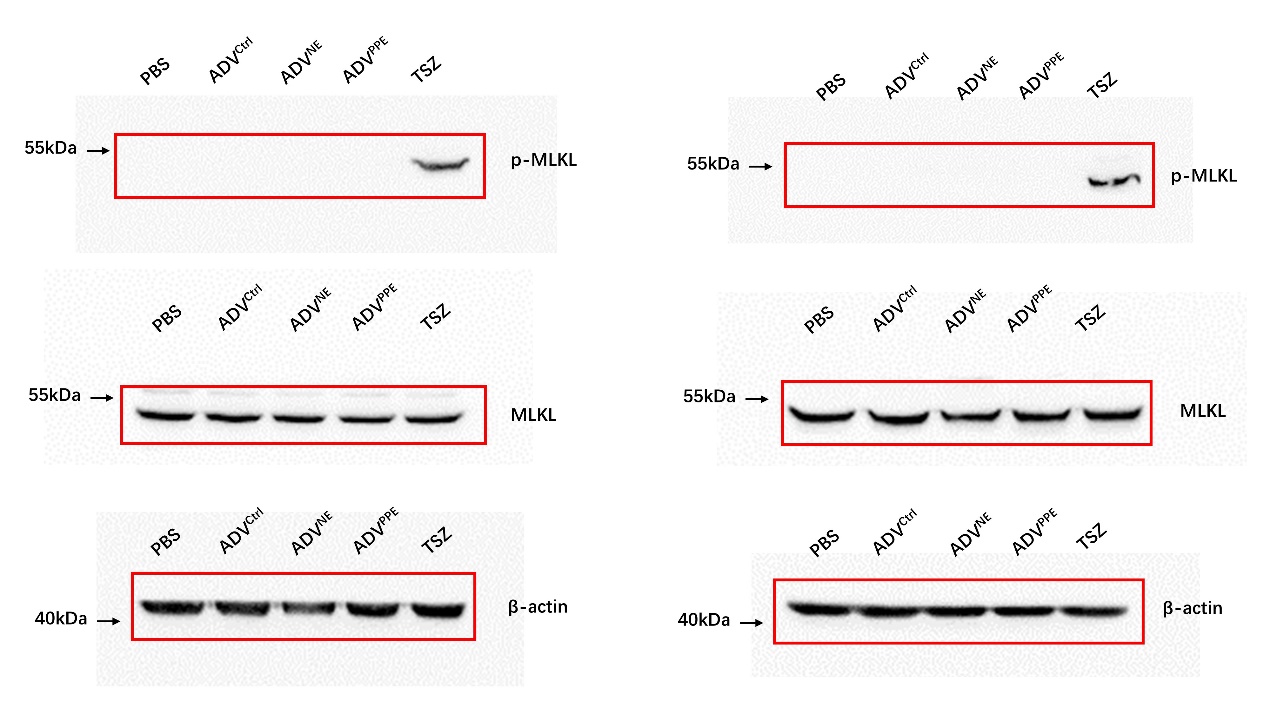


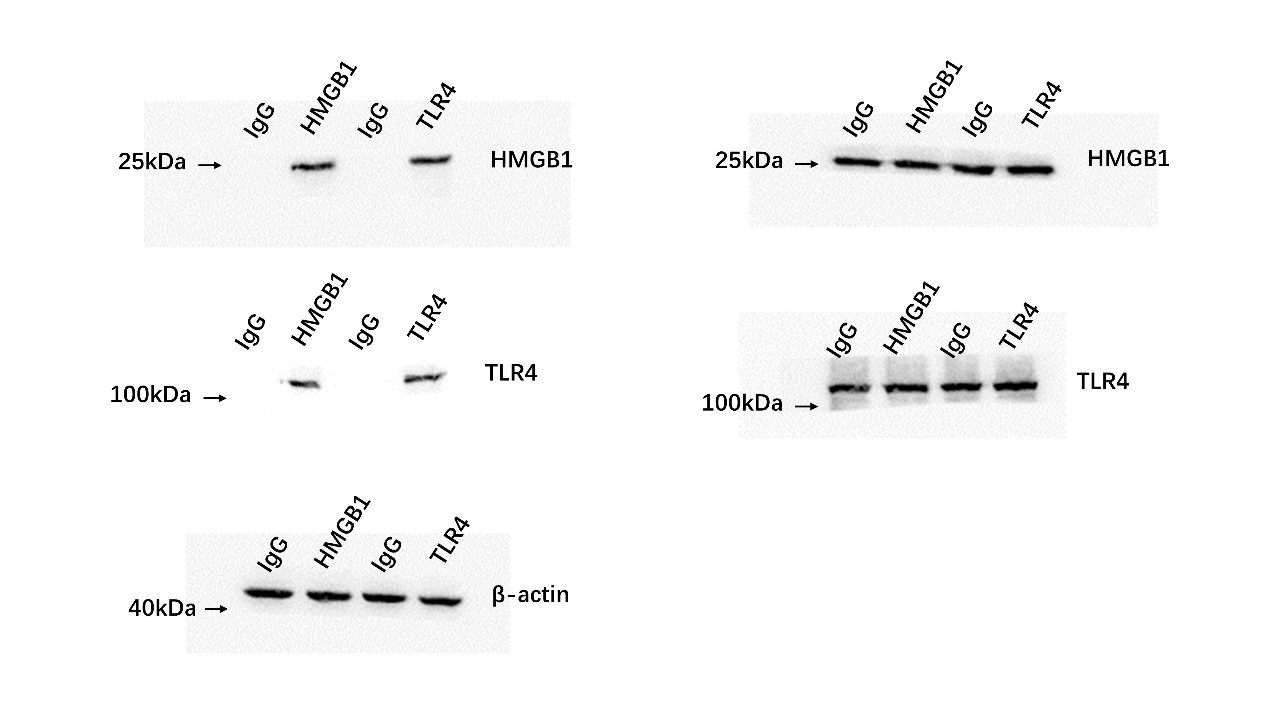


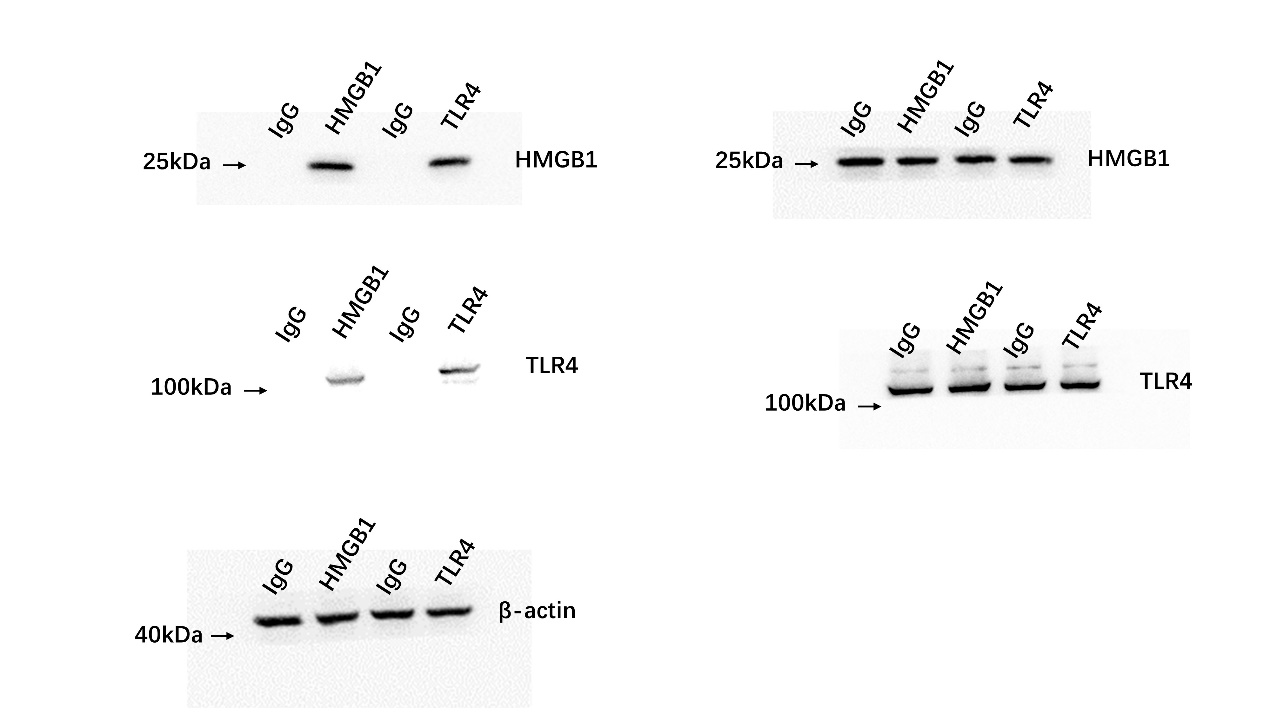


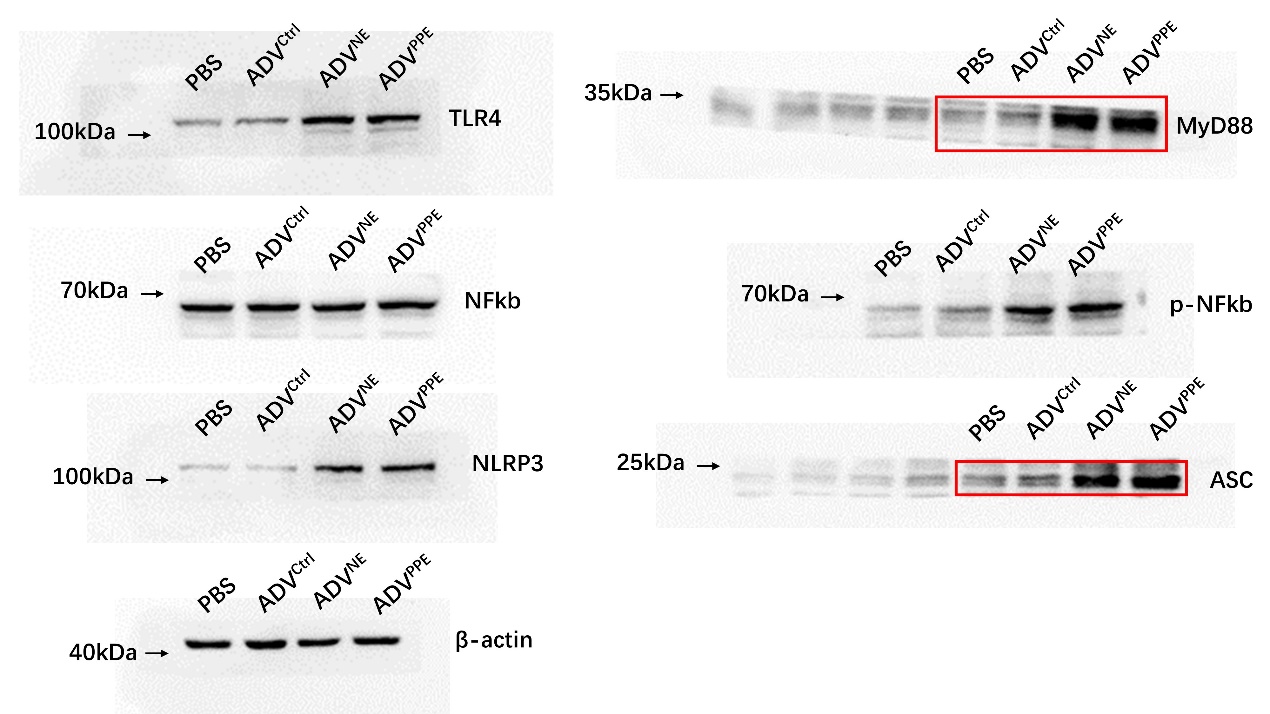


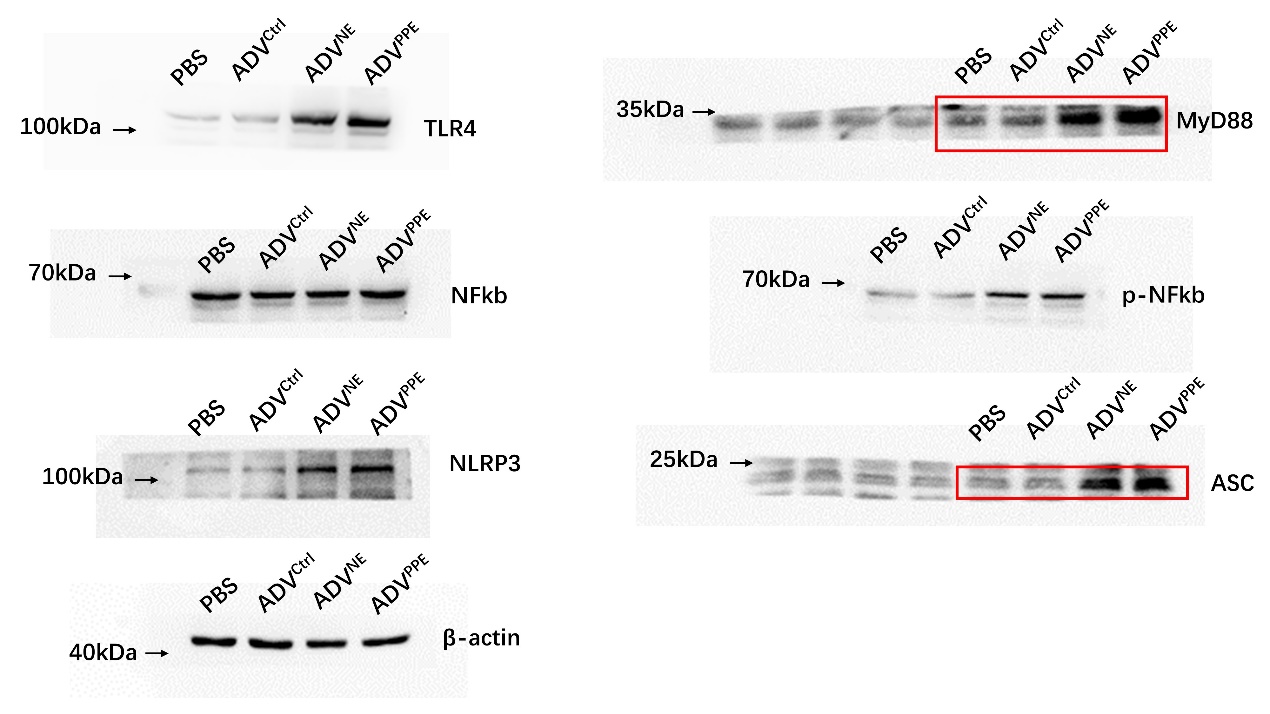


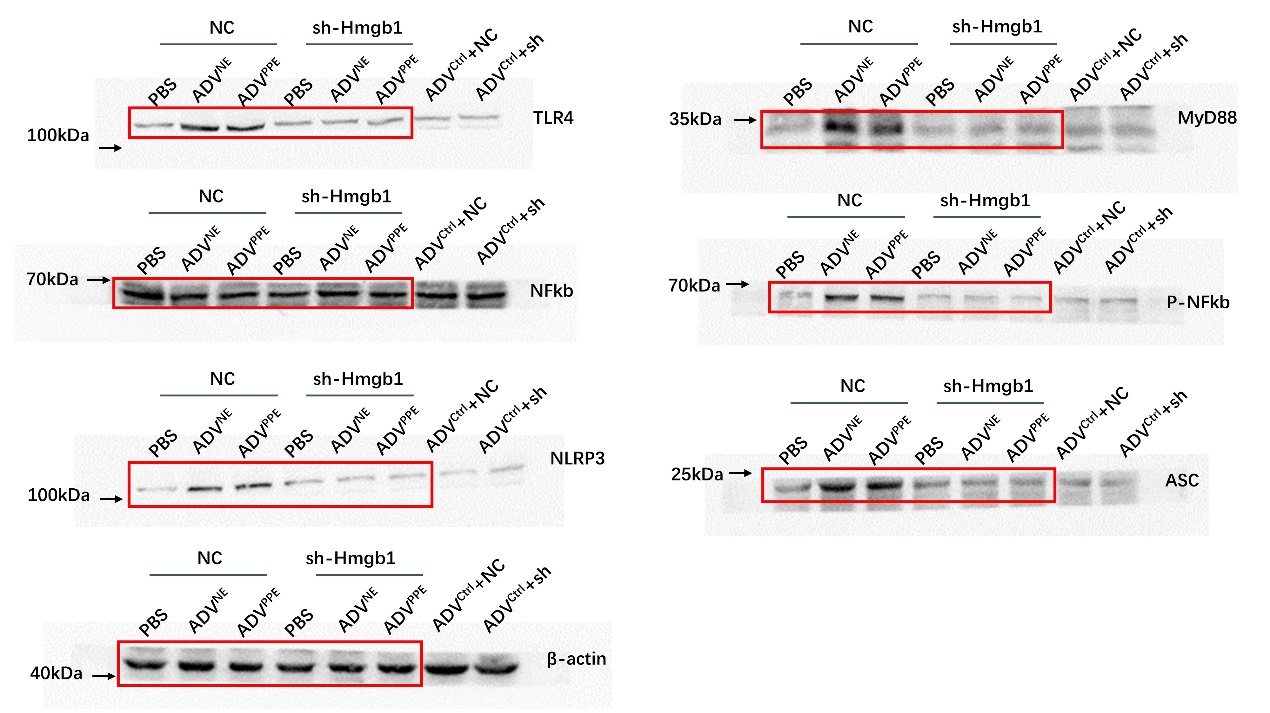


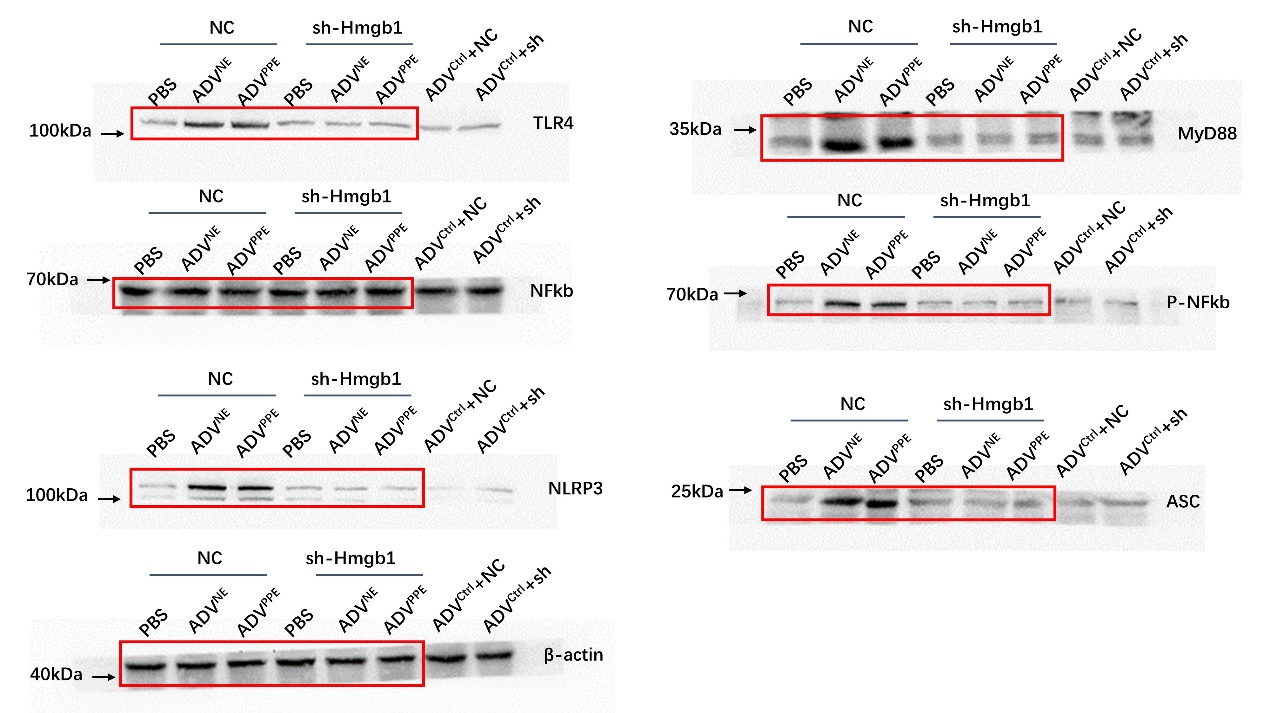

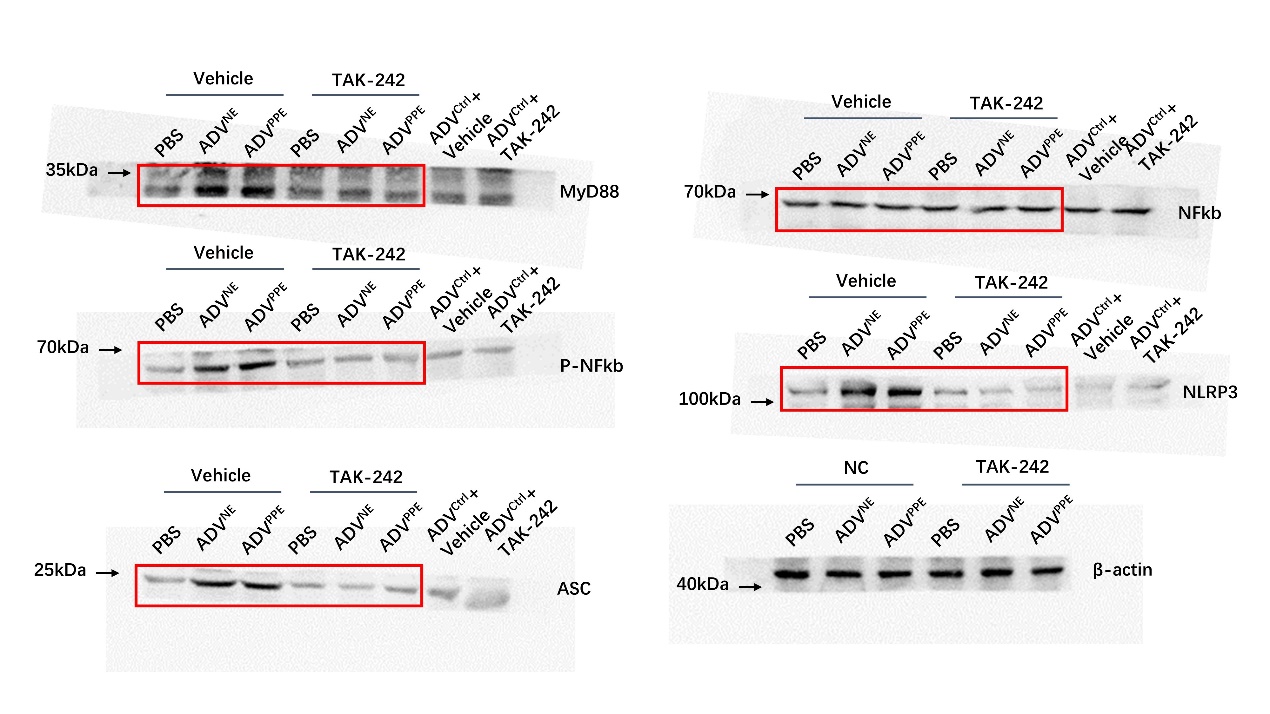

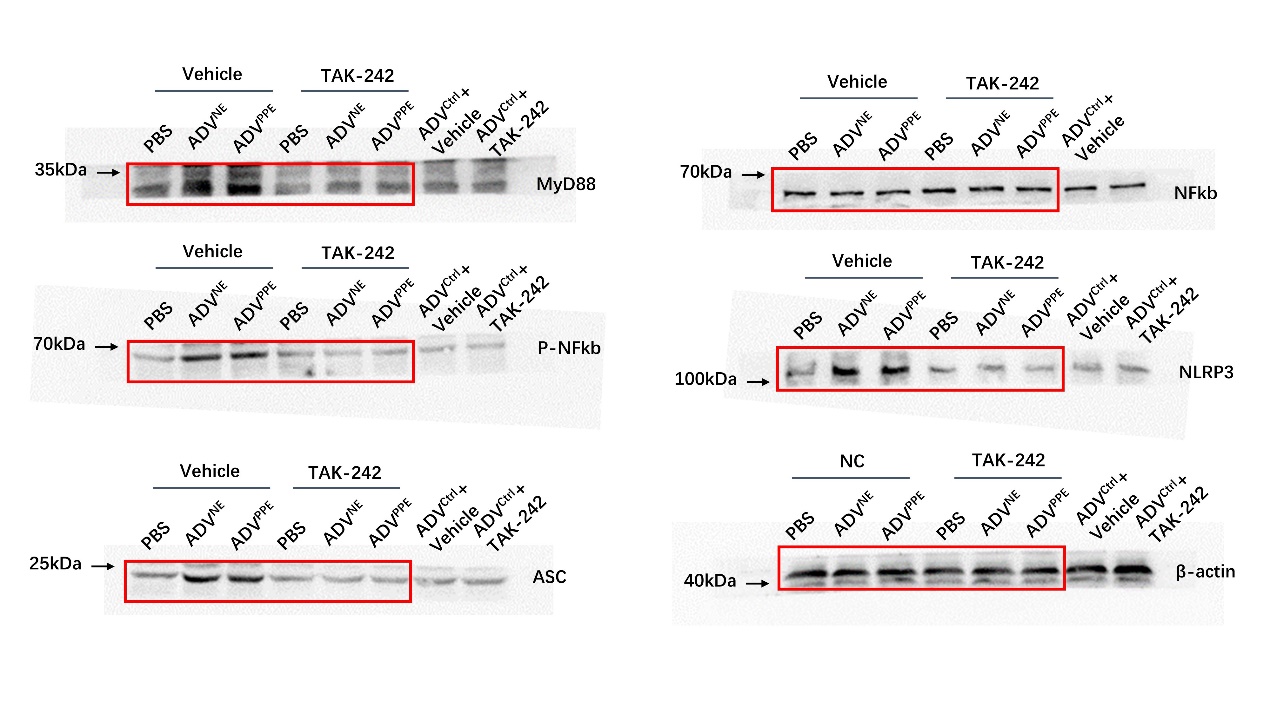


**Figure S7** Uncropped blots.
